# PCGF1-mediated bivalent promoter remodeling enables NK cell immune evasion in non-small cell lung cancer

**DOI:** 10.1038/s41419-026-09003-6

**Published:** 2026-06-30

**Authors:** Hui Zhou, Hechun Lin, Yiru Kong, Hongyu Pan, Wenjun Chai, Lei Sun, Yu Wang, Mengxi Ge, Xiaoyu Ji, Rongrong Yao, Qing Wang, Tao Liu, Mingxia Yan, Xiaohua Liang, Hong Li, Jing Li, Xinli Zhou

**Affiliations:** 1 Department of Oncology, Huashan Hospital Fudan University, Shanghai, China; 2 Department of Oncology, Shanghai Medical College, Fudan University, Shanghai, China; 3 State Key Laboratory of Systems Medicine for Cancer, Shanghai Cancer Institute, Renji Hospital, Shanghai Jiao Tong University School of Medicine, Shanghai, China; 4 Cancer Institute, Fudan University Shanghai Cancer Center, Shanghai, China

**Keywords:** Cancer microenvironment, Cancer epigenetics, Immunoediting, Immune evasion, Non-small-cell lung cancer

## Abstract

Precise regulation of the activating H3K4me3 and repressive H3K27me3 histone modifications at bivalent promoters is essential for normal development but is frequently disrupted in cancer. Among the polycomb group (PCGF) family members, which are key components of polycomb repressive complex 1 (PRC1), PCGF1 emerged as the factor most strongly associated with poor prognosis in non-small cell lung cancer (NSCLC) based on analyses of The Cancer Genome Atlas (TCGA) cohort. In lung cancer cells, PCGF1 upregulation enhanced the deposition of H2AK119ub and H3K27me3 at chromatin. These depositions inhibit the cytokine–cytokine receptor interaction pathway, especially CCL5, CXCL10, CD40, and FAS. Single-cell RNA sequencing further indicated that PCGF1 acts as a negative regulator of natural killer (NK) cell effector function. When NK cell-derived cytokines attempted to activate the cytokine–cytokine receptor interaction pathway in tumor cells, this repressive chromatin state attenuated pathway activation. Consequently, reduced expression of these genes weakened NK cell recruitment and cytotoxic responses. Collectively, this study uncovers a previously unrecognized mechanism by which PCGF1-driven disruption of bivalent promoter balance silences immune signaling cascades, enabling tumor cells to evade NK cell-mediated immunity in NSCLC. These findings highlight bivalent chromatin as a critical regulatory node in tumor immune escape and establish PCGF1 as a promising epigenetic target for immunotherapeutic intervention.

**Figure:**
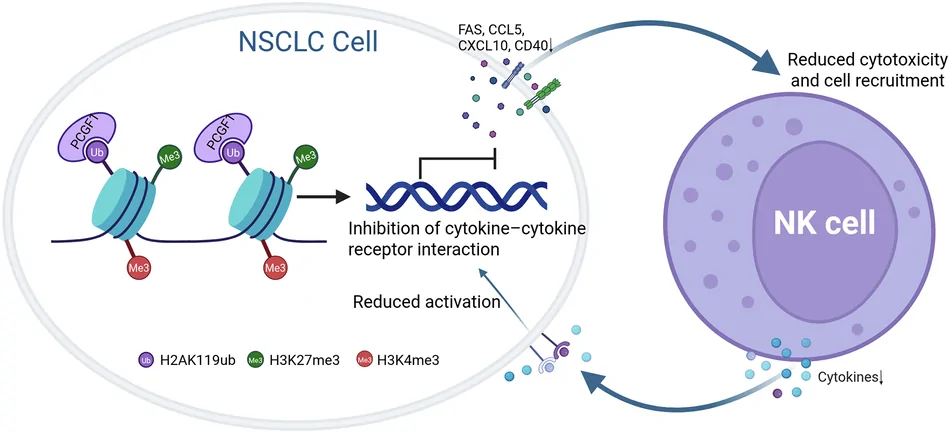
**PCGF1 promotes immune evasion in NSCLC by suppressing cytokine–cytokine receptor interaction pathway**. Upregulation of PCGF1 in NSCLC cells enhances the deposition of the repressive histone modifications H2AK119ub and H3K27me3 while reducing the enrichment of the transcriptionally active histone modification H3K4me3 at target chromatin regions, thereby suppressing cytokine–cytokine receptor interaction pathway. This epigenetic repression reduces the expression of immune-related genes, including CCL5, CXCL10, CD40, and FAS. Consequently, tumor-cell responses to NK cell-derived cytokines are attenuated, leading to impaired NK cell recruitment and cytotoxicity. The schematic diagram was created using BioRender.

**PCGF1 promotes immune evasion in NSCLC by suppressing cytokine–cytokine receptor interaction pathway**. Upregulation of PCGF1 in NSCLC cells enhances the deposition of the repressive histone modifications H2AK119ub and H3K27me3 while reducing the enrichment of the transcriptionally active histone modification H3K4me3 at target chromatin regions, thereby suppressing cytokine–cytokine receptor interaction pathway. This epigenetic repression reduces the expression of immune-related genes, including CCL5, CXCL10, CD40, and FAS. Consequently, tumor-cell responses to NK cell-derived cytokines are attenuated, leading to impaired NK cell recruitment and cytotoxicity. The schematic diagram was created using BioRender.

## Introduction

Immunotherapy is the fourth major treatment modality, alongside surgery, chemotherapy, and radiotherapy in cancer [1]. Immune cell activation, trafficking into the tumor microenvironment (TME), and effector functions depend on a permissive chromatin landscape that can be remodeled to enable dynamic alterations [2, 3]. Epigenetic deregulation is a hallmark of cancer, characterized by pervasive alterations in DNA and histone modifiers [4]. The EZH2 small-molecule inhibitor CPI-205 has been reported to enhance the antitumor effect of CTLA-4 blockade [5]. Notably, emerging evidences indicate that epigenetic therapies not only reprogram tumor-intrinsic transcriptional states but also remodel immune cell activity, redefining epigenetic therapies as potent immune modulators [6–11]. Collectively, these insights highlight the intricate crosstalk between epigenetic regulation and immune response in cancer.

Epigenetic balance at bivalent promoters—marked by activating trimethylation of histone H3 on lysine 4 (H3K4me3) and repressive trimethylation of histone H3 on lysine 27 (H3K27me3)—is crucial for developmental gene regulation and frequently disrupted in cancer [12, 13]. These dual histone marks, deposited by Trithorax (TrxG) and Polycomb (PcG) complexes, maintain genes in a poised configuration that allows dynamic switching between activation and repression. Polycomb repressive complex 2 (PRC2) catalyzes H3K27me3 [14, 15]. Polycomb repressive complex 1 (PRC1), in turn, monoubiquitinates Lys119 of histone H2A (H2AK119ub). Most H2AK119ub deposition arises from non-canonical PRC1 variants containing RYBP or YAF2 and PCGF1, -3, -5, or -6 [16].

PCGF1 (2010002K04Rik/NSPC1/RNF3A-2/RNF68) is a mammalian homolog of the Drosophila polycomb group genes, which act as transcriptional repressors to regulate anterior–posterior patterning in early embryonic development, encoded by a gene located on chromosome 2p13.1. The PRC1.1, composed of PCGF1, targets unmethylated CpG islands to deposit H2AK119ub [17]. This modification promotes PRC2.2 recruitment, which cooperates with PRC2.1 to facilitate H3K27me3 deposition [18]. While these mechanistic insights have been largely derived from embryonic stem cell systems, recurrent mutations and dysregulation of Polycomb proteins across diverse cancers underscore the pathological significance of this axis [19–21].

In NSCLC, CTLA-4 and PD-(L)1 immune checkpoint inhibitors (ICI) have become first-line therapy for patients without actionable oncogenic drivers or with KRAS mutations, but their clinical benefit is limited. In preclinical models of NSCLC, combining 5-Aza and the class I/II histone deacetylase (HDAC) inhibitor entinostat with anti-PD1 and anti-CTLA-4 antibodies has shown enhanced antitumor activity [22]. PCGF1, as a key regulator of bivalently transcribed promoters, is therefore of great importance to investigate its role in immune evasion in NSCLC.

In this study, using cell-based assays, in vivo models, single-cell RNA sequencing, CUT&Tag, ChIP-seq, and bulk RNA-seq, we demonstrate that PCGF1 upregulation suppresses cytokine expression through bivalent promoter remodeling, thereby promoting NK cell immune evasion in NSCLC. These findings identify PCGF1 as a potential therapeutic target and offer new insights for immunotherapy and epigenetic therapy.

## Materials and methods

### Clinical specimens

A total of 63 paired samples of NSCLC tissues and the corresponding adjacent non-tumorous lung tissues were collected from patients who underwent surgical resection at Huashan Hospital, Fudan University. The detailed clinicopathological characteristics of these cases are summarized in Supplementary Table 1. Due to the limited amount of tissue available, protein was successfully extracted from 36 pairs, while RNA was obtained from all 63 pairs. All tissue specimens were immediately snap-frozen in liquid nitrogen after surgical resection and stored in liquid nitrogen until further use. Written informed consent was obtained from all participants prior to sample collection. All experimental procedures involving human tissues were conducted in accordance with the Declaration of Helsinki and approved by the Ethics Committee of Huashan Hospital, Fudan University (approval No. 2022-526) and performed in accordance with the approved experimental protocol and all relevant guidelines and regulations.

### Cell lines and cell culture

All cell lines used in this study were obtained from the American Type Culture Collection (ATCC, Manassas, VA, USA), except for primary mouse NK cells and NK-92MI cells. Lewis lung carcinoma (LLC) cells (ATCC, CRL-1642) were cultured in Dulbecco’s modified Eagle medium (DMEM; Basal Media, L110KJ, Shanghai, China) supplemented with 10% fetal bovine serum (FBS; Gibco, A5670701, Grand Island, NY, USA) and 1% penicillin–streptomycin solution (Basal Media, S110JV). Human lung carcinoma A549 cells (ATCC, CCL-185) were maintained in Ham’s F-12K medium (Basal Media, L450KJ) containing 10% FBS and 1% penicillin–streptomycin (Basal Media, S110JV). Both cells were authenticated by STR profiling before use. Primary mouse NK cells were cultured in RPMI-1640 medium (Basal Media, L220KJ) supplemented with 10% FBS, 1% penicillin-streptomycin, 1% non-essential amino acids (Beyotime, C0332, Shanghai, China), 1% sodium pyruvate (Beyotime, C0331), 0.1 mM 2-mercaptoethanol (MCE, HY-Y0326, Shanghai, China), and recombinant mouse IL-15 (10 ng/mL; MCE, HY-P78719). The NK-92MI cell line was obtained from the National Collection of Authenticated Cell Cultures (NCACC, GNHu22, Shanghai, China) and maintained in the supplier-recommended complete medium (NCACC, MD105). All cells were maintained at 37 °C in a humidified atmosphere containing 5% CO₂. All experiments were performed using cells at passages below 20. Mycoplasma contamination was monitored weekly using a Mycoplasma Detection Kit (Yeasen, 40601ES20, Shanghai, China), and only mycoplasma-negative cells were used in subsequent experiments.

### Isolation of primary murine NK cells

Primary NK cells were isolated from the spleens of 8–12-week-old C57BL/6J mice. To activate NK cells in vivo, mice were intraperitoneally injected with 300 µg poly(I:C) LMW (InvivoGen, tlrl-picw, Waltham, MA, USA) dissolved in 200 µL DPBS using an insulin syringe 18–24 h before sacrifice [23]. Spleens were mechanically dissociated and passed through a 70 µm cell strainer (JET Biofil, CSS013070 Guangzhou, Guangdong, China) to obtain single-cell suspensions, followed by red blood cell lysis (Beyotime, C3702). NK cells were enriched by negative magnetic selection using the EasySep™ Mouse NK Cell Isolation Kit (STEMCELL Technologies, 19855, Vancouver, BC, Canada) according to the manufacturer’s instructions.

### Flow cytometry-based NK cell cytotoxicity assay

Primary murine NK cells and human NK-92MI cells were used as effectors, while LLC and A549 cells served as target cells for mouse and human assays, respectively. For the ex vivo murine NK cell assay, enriched NK cells were resuspended in complete NK medium (2 × 10⁶ cells/mL) and seeded into 96-well (JET Biofil, TCP011096) plates at the indicated effector-to-target (E:T) ratios (5:1, 2:1, 1:1, and 0.5:1). Wild-type (LLC WT) and Pcgf1-knockout (LLC Pcgf1 KO) cells were labeled using the CFDA SE Cell Proliferation and Tracking Kit (Beyotime, C0051) following the manufacturer’s instructions and co-cultured with NK cells for 4 h at 37 °C. For the human NK-92MI assay, NK-92MI cells were cultured in complete NK-92MI medium. Wild-type (A549 WT) and PCGF1-knockout (A549 PCGF1 KO) cells were labeled with CFDA SE under identical conditions and co-cultured with NK-92MI cells at the indicated E:T ratios for 18 h at 37 °C. Cell death was assessed using Zombie NIR™ Fixable Viability Dye (1:500; BioLegend, 423106, San Diego, CA, USA) according to the manufacturer’s instructions. Samples were analyzed on a BD LSR Fortessa X-20 cytometer (BD Biosciences, San Jose, CA, USA), and NK cytotoxicity was quantified as the percentage of Zombie NIR⁺ target cells among CFDA SE⁺ populations.

### Treatment of LLC cells with NK cell-conditioned medium

Activated primary NK cells isolated from C57BL/6J mice spleens were cultured in complete NK medium for 24 h. The culture supernatant was collected by centrifugation at 400×*g* for 5 min, and 15 mL of the clarified supernatant per sample was transferred into Amicon® Ultra centrifugal filter units (3 kDa MWCO; Millipore, UFC900396, Burlington, MA, USA). The samples were centrifuged at 4000×*g* for 30 min at 4 °C to concentrate the NK cell-conditioned medium. The concentrated NK supernatant was then mixed with complete DMEM at a ratio of 1:2 and applied to LLC WT and Pcgf1 KO LLC cells. After 12 h of incubation, the supernatant was removed, the cells were washed three times with PBS (Basal Media, B310KJ), and lysed directly in TRIzol reagent (Beyotime, R0016) for subsequent bulk RNA-seq analysis.

### Bulk RNA sequencing

Total RNA was extracted using TRIzol reagent according to the manufacturer’s instructions. RNA purity and concentration were determined using a NanoDrop 2000 spectrophotometer (Thermo Fisher Scientific, Waltham, MA, USA), and RNA integrity was assessed with an Agilent 2100 Bioanalyzer (Agilent Technologies, Santa Clara, CA, USA).

RNA-seq libraries were constructed using the VAHTS Universal V8 RNA-seq Library Prep Kit for Illumina (Vazyme, NR605-01, Nanjing, Jiangsu, China) following the manufacturer’s protocol. Briefly, mRNA was enriched from total RNA using VAHTS mRNA Capture Beads 2.0 and then fragmented under heat conditions. Fragmented mRNA was used for first-strand cDNA synthesis, followed by second-strand cDNA synthesis. The resulting double-stranded cDNA was subjected to adapter ligation after end repair and A-tailing, and the ligated products were purified using VAHTS DNA Clean Beads. Libraries were then PCR-amplified, followed by an additional bead-based purification step. Library quality and fragment size distribution were evaluated using an Agilent 2100 Bioanalyzer, and library concentration was determined using Qubit fluorometric quantification and qPCR prior to sequencing. Qualified libraries were sequenced on an Illumina platform using a paired-end 150 bp strategy. Transcriptome sequencing and subsequent bioinformatic analyses were performed by OE Biotech Co., Ltd. (Shanghai, China). Three biological replicates were analyzed for each group.

### RNA sequencing and differential gene expression analysis

RNA-seq libraries were sequenced on the Illumina NovaSeq 6000 platform (Illumina, San Diego, CA, USA) to generate 150 bp paired-end reads, yielding ~28 million raw reads per sample. Raw sequencing reads in FASTQ format were processed using fastp [24] to remove adapter sequences and low-quality reads, resulting in high-quality clean reads for downstream analysis. Clean reads were aligned to the *Mus musculus* reference genome (mm39) using HISAT2 [25]. Gene expression levels were quantified as fragments per kilobase of transcript per million mapped reads (FPKM) [26], and raw read counts for each gene were obtained using HTSeq-count [27]. Principal component analysis (PCA) and visualization were performed in R (v3.2.0) to assess sample reproducibility among biological replicates. Differentially expressed genes (DEGs) were identified using the DESeq2 [28] package in R. Genes with q < 0.05 and |log₂(fold change)| > 0.5 were defined as significantly differentially expressed. Hierarchical clustering of DEGs was conducted to visualize expression patterns across groups, and radar plots of the top 30 upregulated and downregulated genes were generated using the ggradar package. Functional enrichment analyses of DEGs were performed using hypergeometric testing based on the Gene Ontology (GO) [29], Kyoto Encyclopedia of Genes and Genomes (KEGG) [30], Reactome, and WikiPathways databases to identify significantly enriched biological processes and pathways. Enrichment results were visualized as bar plots, chord diagrams, or circular enrichment maps in R (v3.2.0). Gene set enrichment analysis (GSEA) [31, 32] was performed to evaluate the enrichment of predefined gene sets ranked according to differential expression between the indicated groups.

### Generation of PCGF1 knockout cell lines

To disrupt PCGF1 expression, CRISPR/Cas9-mediated genome editing was performed in both mouse LLC and human A549 cells.

For LLC cells, the sgRNA sequence (5′-AAGGACATACCTTCACCACT-3′), designed using the CRISPOR web tool, targeted exon 7 of PCGF1. The sgRNA was cloned into the lentiCRISPRv2 vector (Addgene, 52961, Watertown, MA, USA). Lentiviral particles carrying the sgRNA/Cas9 construct were generated in packaging cells using Lipofectamine 3000 (Thermo Fisher Scientific, L3000015) and subsequently used to transduce LLC cells. After 3–4 days, cells were subjected to limiting dilution in 96-well plates to obtain single-cell-derived clones. Individual colonies were expanded, and genomic DNA was extracted from each clone. The genomic region spanning the sgRNA target site was amplified by PCR using primers flanking the edited locus (forward, 5′-AGGGACCTGAGCATCTTCGTT-3′; reverse, 5′-TAGCATCTGGGTCACCAGTAGTA-3′). PCR products were examined by agarose gel electrophoresis, purified, and subjected to Sanger sequencing (GENEWIZ, Suzhou, Jiangsu, China). Sequencing chromatograms were aligned to the wild-type reference sequence to confirm the presence of CRISPR-induced indels at the expected cleavage site. A clone carrying a single-nucleotide deletion in exon 7, which caused a frameshift and introduced a premature stop codon, was selected as the PCGF1 knockout LLC clone. Loss of PCGF1 protein expression was further confirmed by Western blot.

For A549 cells, PCGF1 knockout cells were generated by Ubigene Biosciences (Guangzhou, China) using CRISPR/Cas9-based genome editing. The sgRNA sequence (5′-GCAGGACATCGTGTATAAGC-3′) targeting exon 7 of PCGF1 was introduced together with Cas9 by electroporation. Single-cell clones were isolated by limiting dilution and expanded for validation. Genomic DNA was extracted from candidate clones, and the targeted region was amplified by PCR using the following primers: forward, 5′-TGCGCCGGCTACTTCGT-3′; reverse, 5′-GGCTCTGAGAAAGATAGGGTAGTG-3′. PCR amplicons were purified and analyzed by Sanger sequencing to verify editing at the target locus. A clone carrying a 98 bp deletion in exon 7 was identified and selected as the PCGF1 knockout A549 clone. The absence of PCGF1 protein expression was further confirmed by Western blot.

### Generation of PCGF1 rescue cell lines

To restore Pcgf1/PCGF1 expression after knockout, codon-optimized Pcgf1/PCGF1 coding sequences fused to an HA tag were synthesized and cloned into the pGLV58 lentiviral expression vector under the control of the CMV promoter. The pGLV58 backbone contains the lentiviral elements 5′ LTR, HIV-1 ψ, RRE, cPPT/CTS, WPRE, and 3′ LTR (ΔU3), as well as NeoR/KanR and AmpR selection markers. Recombinant plasmids were verified by Sanger sequencing and used for lentivirus production. The resulting lentiviral particles were used to infect Pcgf1-KO LLC cells and PCGF1-KO A549 cells, followed by antibiotic selection to establish stable rescue cell lines. Cells infected with the empty vector were used as KO Vector controls, whereas cells re-expressing Pcgf1/PCGF1 were designated KO Rescue. Re-expression efficiency was confirmed by western blot using antibodies against Pcgf1/PCGF1 and HA. The codon-optimized coding sequences are provided in the Supplementary File.

### In vivo NK cell depletion

To deplete NK cells in vivo, mice were treated with anti-NK1.1 antibody (clone PK136) (InVivoCrown, IVC-IV0111, Cambridge, UK) by intraperitoneal injection. Each mouse received 300 μg antibody per injection, administered once every 3 days during the course of the experiment. The antibody was prepared in PBS and injected intraperitoneally at 200 μL. Mice in the control group were treated with the corresponding isotype control (InVivoCrown, IV0137). NK cell depletion efficiency was confirmed by flow cytometric analysis of splenic NK cells 24 h after antibody injection.

### Protein extraction

Total protein was extracted from both tissue samples and cultured cells using the Tissue Protein Extraction Kit (Beyotime, P0012) according to the manufacturer’s instructions.

For tissue samples, frozen tissues were ground thoroughly in liquid nitrogen using a prechilled mortar and pestle. The resulting powder was lysed in the extraction buffer, and the homogenate was transferred to a spin column fitted with a collection tube and centrifuged at 12,000×*g* for 30–60 s at 4 °C. The spin column was discarded, and the flow-through collected in the tube was used as the total protein lysate. For cultured cells, after removing the culture medium, cells were washed twice with PBS or physiological saline. Lysis buffer was then added at 100–200 µL per well of a six-well plate (JET Biofil, TCP011006), and the cell layer was gently pipetted several times to ensure complete mixing. After incubation on ice for 10 min, the lysate was centrifuged at 12,000×*g* for 30–60 s at 4 °C, and the resulting supernatant was collected as the total protein sample for downstream analyses.

### Nuclear protein extraction

Nuclear proteins were extracted using the Nucleoprotein Extraction Kit (Sangon Biotech, C500009, Shanghai, China) according to the manufacturer’s instructions, with all procedures performed on ice or at 4 °C. For adherent cells, the culture medium was aspirated, and the cells in 150 mm dishes (JET Biofil, TCD110150) were washed twice with 10 mL of prechilled 1× PBS. After gentle agitation, the residual buffer was removed completely. A fresh hypotonic buffer was prepared by supplementing each 1 mL of prechilled Hypotonic Buffer with 5 µL phosphatase inhibitor, 10 µL PMSF, and 1 µL DTT. Cells were resuspended in the prepared buffer and collected into prechilled tubes using a cell scraper (for adherent cultures). The suspension was gently flicked to resuspend the pellet and incubated on ice for 10 min, followed by vortexing for 10 s. The mixture was centrifuged at 800×*g* for 5 min at 4 °C, and the supernatant was discarded. The pellet was washed with 0.4 mL of prechilled Hypotonic Buffer for 30 s, centrifuged again at 2500×*g* for 5 min, and the supernatant was removed. The final pellet was resuspended in 0.2 mL of prechilled Lysis Buffer (supplemented with 5 µL phosphatase inhibitor, 10 µL PMSF, and 1 µL DTT per 1 mL buffer) and incubated on ice for 20 min. The lysate was centrifuged at 20,000×*g* for 10 min at 4 °C, and the resulting supernatant containing nuclear proteins was collected, aliquoted, and stored at −80 °C until use. Repeated freeze–thaw cycles were avoided.

### Histone extraction

Cells were washed twice with 1× PBS, and the supernatant was completely removed after each wash. NETN buffer consisted of 0.5% NP-40 (Beyotime, P0013F), 20 mM Tris–HCl (pH 8.0; Beyotime, ST780), 500 mM NaCl (Beyotime, ST347), and 1 mM EDTA (Beyotime, C0196) in ddH₂O, supplemented with protease inhibitor cocktail (Beyotime, P1006) immediately before use. NETN lysis buffer was then added, and the cells were lysed on ice for 15 min. Cells were scraped and collected into 1.5 mL microcentrifuge tubes, followed by centrifugation at 1500×*g* for 10 min at 4 °C. The pellet was then washed once or twice with 1 mL of prechilled NETN buffer under the same centrifugation conditions to remove residual non-histone proteins and detergent-soluble components. After complete removal of the NETN supernatant, the pellet was resuspended in 100 µL of 0.2 M HCl and incubated on ice for 30 min for acid extraction of histones. Samples were then centrifuged at 12,000×*g* for 15 min at 4 °C, and the supernatant containing solubilized histones was transferred to a new tube. The acidic extracts were neutralized by adding 20 µL of 1 M Tris–HCl (pH 8.0) per 100 µL of HCl extract. Neutralized histone samples were aliquoted and stored at −80 °C until use.

### Western blot analysis

Protein samples extracted as described above were separated by SDS–polyacrylamide gel electrophoresis (SDS–PAGE) and transferred onto 0.22 µm PVDF membranes (Millipore, SEQ00010). The membranes were blocked with 5% skim milk for 1 h at room temperature and incubated with primary antibodies overnight at 4 °C. The following primary antibodies were used: anti-PCGF1 (1:1000; Abcam, ab183499, Cambridge, UK), anti-H3K4me3 (1:1000; Cell Signaling Technology, 9751, Danvers, MA, USA), anti-H3K27me3 (1:1000; Cell Signaling Technology, 9733), anti-H2AK119ub (1:2000; Cell Signaling Technology, 8240), anti-Histone H3 (1:5000; Proteintech, 17168-1-AP, Wuhan, Hubei, China), anti-HA (1:5000; Proteintech 51064-2-AP), anti-β-Actin (1:5000, Proteintech 66009-1-Ig) and anti-Lamin B1 (1:5000; Proteintech 12987-1-AP). After washing, the membranes were incubated with HRP-conjugated goat anti-rabbit secondary antibody (1:5000; Proteintech, SA00001-2) for 2 h at room temperature. Protein bands were visualized using Immobilon™ Western Chemiluminescent HRP Substrate (Millipore).

### Transwell migration assay

Tumor cells were seeded in six-well plates at 2.5 × 10^6^ cells per well and allowed to adhere overnight. The next day, the culture medium was replaced with 2 mL serum-free DMEM, and cells were incubated for 6 h. The conditioned medium was then collected and centrifuged to remove residual tumor cells. For the migration assay, 600 μL of conditioned medium was added to the lower chamber of a 24-well plate. Transwell inserts containing a polycarbonate membrane (5 μm pore size, Corning, 3421, NY, USA) were placed into the wells. NK cells (5 × 10^5^ cells in 100 μL serum-free medium) were added to the upper chamber and incubated at 37 °C for 2 h. After incubation, cells that had migrated into the lower chamber were collected, mixed with counting beads (BioLegend, 424902), and analyzed by flow cytometry to quantify the number of migrated cells.

### Animal experiments

All animal experiments were conducted in accordance with the ARRIVE 2.0 guidelines and approved by the Institutional Animal Care and Use Committee (IACUC) of Fudan University (approval Nos. FUSCC-IACUC-2025175; FUSCC-IACUC-2025059; FUSCC-IACUC-2024668) and performed in accordance with the approved experimental protocol and all relevant guidelines and regulations. Male C57BL/6J mice, male Rag1^−/−^ mice, and male BALB/c nude mice (4–6 weeks old, ~18–22 g) were purchased from GemPharmatech Co., Ltd. (Nanjing, Jiangsu, China). All mice were maintained in a specific pathogen-free (SPF) facility under standard conditions (12 h light/dark cycle, 22 °C, 60% humidity) with ad libitum access to standard chow and sterile water. Mice were acclimated for 7 days before experimentation. Control and experimental groups were housed, treated, and analyzed simultaneously within the same experimental batch under identical conditions.

No statistical method was used to predetermine sample size. Sample sizes were chosen based on our previous experience, preliminary experiments, and common practice in the field. No animals were excluded unless a predefined technical failure occurred. Mice were randomly assigned to experimental groups before tumor inoculation or treatment. Investigators were not blinded to group allocation during the experiments or outcome assessment unless otherwise stated. All animal experiments were conducted in accordance with the ethical standards of the Institutional Animal Care and Use Committee (IACUC) of Fudan University. The maximal tumor size permitted by the IACUC was 2 cm in diameter or 2000 mm³ in volume, and no tumors exceeded these limits during in vivo monitoring. Minor discrepancies between ex vivo tumor images and in vivo measurements were attributable to normal biomechanical changes, including tissue relaxation and morphological alterations after dissection. Source data for the tumor growth curves are provided in Supplementary Table 2 in accordance with the journal’s data availability policy.

For subcutaneous tumor models, 1 × 10⁶ LLC cells or 1 × 10⁶ A549 cells suspended in 200 μL PBS were injected into the right axillary region of each mouse. LLC cells were implanted into C57BL/6J mice and Rag1^−/−^ mice, whereas A549 cells were implanted into BALB/c nude mice. WT or empty vector-transduced cells served as controls in each experiment and were compared with the corresponding PCGF1-manipulated cells.

Tumor growth was monitored every 3 days using calipers, and tumor volume was calculated as follows: volume = length × width²/2. At the designated experimental endpoints, mice were euthanized, tumors were collected and measured, and tumor-infiltrating immune cells were analyzed by flow cytometry. The experimental unit was one mouse.

### Flow cytometry analysis of tumor-infiltrating immune cells

Subcutaneous tumors were excised from the axillary region of mice and finely minced into small fragments. The tissue was enzymatically dissociated in a digestion buffer containing Collagenase IV (Sigma, C4-22-1G, St. Louis, MO, USA) and DNase I (Sigma, 10104159001). The resulting cell suspension was passed through a 70 µm cell strainer to remove debris, followed by treatment with red blood cell lysis buffer (Beyotime, C3702). After washing, cells were incubated with fluorochrome-conjugated antibodies for surface staining. For intracellular staining, cells were fixed and permeabilized according to the manufacturer’s protocol. Flow cytometric analysis was performed on a BD LSR Fortessa X-20 cytometer, and data were analyzed using FlowJo software (version 10.8.1). The list of antibodies used for flow cytometry is provided in Supplementary Table 3.

### ChIP-seq

Chromatin immunoprecipitation followed by sequencing (ChIP-seq) was performed by Shanghai Biotechnology Corporation (Shanghai, China). Briefly, cells were cross-linked with 1% formaldehyde (Sinopharm Chemical Reagent Co., Ltd, 10010061, Shanghai, China) for 10 min at room temperature, and the reaction was quenched with 0.125 M glycine (Beyotime, ST085) for 5 min. Cells were then lysed, and nuclei were collected by centrifugation and further processed to release chromatin. Chromatin was fragmented by sonication (10 s on, 30 s off, for 20 cycles) to obtain DNA fragments of ~200–500 bp. After fragmentation, 10% of the chromatin was reserved as the input control, 80% was subjected to immunoprecipitation with an anti-Pcgf3/5 antibody (1:50; Abcam, ab201510) overnight at 4 °C on a rotating platform for immunoprecipitation. Following immunoprecipitation, cross-links were reversed, and DNA was purified by phenol–chloroform extraction.

Sequencing libraries were prepared by end repair, adapter ligation, and PCR amplification. PCR-amplified libraries were purified using 1.3× magnetic beads. Library fragment size distribution was assessed using an Agilent 2100 Bioanalyzer (Agilent Technologies), and library concentration was determined using the Quant-iT™ dsDNA HS Assay Kit (Invitrogen, MA, USA) in combination with qPCR. Qualified libraries were sequenced on an Illumina platform using a paired-end 150 bp strategy, generating at least 6 Gb of raw data per sample for subsequent bioinformatic analysis. One biological replicate library was generated for each group.

### CUT&Tag

Cleavage Under Targets and Tagmentation (CUT&Tag) libraries were prepared using the Hyperactive Universal CUT&Tag Assay Kit for Illumina (Vazyme, TD903-01) according to the manufacturer’s instructions. All buffers were freshly prepared on the day of use, and digitonin-containing solutions were kept on ice. Briefly, 5 × 10^4^ cells per sample were washed and bound to ConA-coated magnetic beads. Bead-bound cells were incubated with the indicated primary antibodies overnight at 4 °C, followed by incubation with the appropriate secondary antibody for 1 h at room temperature. Samples were then incubated with pA/G-Tn5 transposome for 1 h at room temperature. After washing, tagmentation was carried out at 37 °C for 1 h. The reaction was stopped by adding SDS, and DNA was recovered using magnetic beads.

CUT&Tag libraries were PCR-amplified and purified using VAHTS DNA Clean Beads (Vazyme, N411). Library quality was assessed by Qubit fluorometric quantification and qPCR-based library quantification prior to sequencing. Sequencing was carried out on an Illumina NovaSeq 6000 platform with paired-end 150 bp reads by OBiO Biotech (Shanghai, China). The following primary antibodies were used for tagmentation: anti-H3K4me3 (1:50; Cell Signaling Technology, 9751), anti-H3K27me3 (1:50; Cell Signaling Technology, 9733), and anti-H2AK119ub (1:50; Cell Signaling Technology, 8240). For CUT&Tag experiments, three biological replicate libraries were prepared for each control and experimental group.

### Promoter and CpG island annotation

Genomic coordinates of CpG islands were downloaded from the UCSC Genome Browser database [33]. Promoter regions were defined as ±2 kb surrounding the transcription start site (TSS) of the longest annotated transcript for each gene based on Ensembl release 67. Promoters marked by both H3K4me3 and H3K27me3 were classified as bivalent promoters, those marked only by H3K4me3 were defined as active promoters, and those marked only by H3K27me3 were defined as repressed promoters. Promoters overlapping CpG islands by at least 1 bp were designated as CGI promoters, whereas promoters without CpG island overlap were designated as non-CGI promoters [34].

### ChIP-seq data analysis

ChIP-seq libraries were sequenced on an Illumina platform using a paired-end 150 bp strategy. Raw sequencing reads were first processed to remove adapter sequences and low-quality bases using Trim Galore (v0.6.11) with default parameters. Clean reads were then aligned to the mouse reference genome (mm39) using Bowtie2 (v2.3.4.4) with the following parameters: --end-to-end --no-mixed --no-discordant --no-unal --phred33 -I 10 -X 2000 [35]. The resulting SAM files were converted to BAM format, sorted, and indexed using SAMtools (v1.3.1) [36].

PCR duplicates were removed using the SAMtools markdup workflow. Briefly, BAM files were sorted by read name, mate information was fixed using samtools fixmate, reads were coordinate-sorted, and duplicate reads were removed using samtools markdup -r. Only uniquely mapped, non-duplicate reads were retained for downstream analysis.

For peak calling, MACS2 (v2.2.7.1) was used with the corresponding input sample as control and the following parameters: -f BAMPE -g mm -q 0.01 --keep-dup all --call-summits [37]. Peaks were annotated using ChIPseeker (v1.40.0). Genome-wide coverage tracks were generated from duplicate-removed BAM files using bamCoverage in deepTools with CPM normalization and a bin size of 10 bp, and were visualized in IGV. For enrichment heatmaps, signal matrices were generated using the computeMatrix function in deepTools (v3.2.1) and displayed using plotHeatmap [38]. For visualization of ChIP enrichment, the input signal was used as a control.

### CUT&Tag data analysis

CUT&Tag libraries were sequenced on an Illumina NovaSeq 6000 platform using a paired-end 150 bp strategy. Raw reads were aligned to the mouse reference genome (mm39) using Bowtie2 (v2.3.4.4) with the following parameters: --end-to-end --very-sensitive --no-mixed --no-discordant --phred33 -I 10 -X 700 [35]. PCR duplicates were removed with SAMtools (v1.3.1) [36], and only properly paired mapped reads were retained for downstream analysis. To estimate spike-in depth, the same reads were also aligned to the lambda genome using the same Bowtie2 parameters.

Mapped BAM files were converted to BEDPE format using bedtools bamtobed, and read pairs mapping to the same chromosome with fragment lengths <1000 bp were retained. Fragment BED files were then generated and used for genome-wide coverage analysis. For spike-in normalization, sequencing depth derived from lambda-aligned reads was used to calculate a scaling factor (10,000/spike-in depth), and normalized bedGraph tracks were generated using bedtools genomecov.

Peak calling was performed using SEACR (v1.3) in non-stringent mode with a threshold of 0.1. CPM-normalized bigWig files were generated from sorted BAM files using bamCoverage for visualization in IGV.

For differential peak analysis, peaks identified from all samples were merged to generate a master peak set using the reduce function in GenomicRanges (v1.56.1). Fragment counts within each merged peak were quantified from BAM files using the getCounts function in chromVAR (v1.26.0) (paired = TRUE). Peaks with a total count ≤ 5 across all samples were excluded. Differential enrichment analysis was then performed using DESeq2 (v1.44.0) with the design formula ~ condition [28]. Peaks with an absolute log2 fold change >1 and an adjusted *P* value < 0.05 were considered significantly differentially enriched.

### RNA extraction and quantitative real-time PCR (RT-qPCR)

Total RNA was extracted using TRIzol reagent according to the manufacturer’s protocol. Briefly, cells were incubated with TRIzol for 10 min at room temperature, followed by the addition of chloroform (Sinopharm Chemical Reagent Co., Ltd, SUP1024321000) and vigorous shaking for 10 s. After standing for 10 min, samples were centrifuged at 12,000×*g* for 10 min at 4 °C, and the upper aqueous phase was carefully transferred to a new RNase-free tube (Thermo Fisher Scientific, 509-GRD-Q). RNA was precipitated by adding an equal volume of isopropanol, incubating the mixture on ice for 10 min, and centrifuging again at 12,000×*g* for 10 min at 4 °C. The RNA pellet was washed twice with 75% ethanol (Sinopharm Chemical Reagent Co., Ltd, 80176961), air-dried, and dissolved in RNase-free water (Thermo Fisher Scientific, AM9906) before storage at −80 °C. Quantitative real-time PCR was carried out on a QuantStudio 6 Flex Real-Time PCR System (Thermo Fisher Scientific) using SYBR Green Master Mix (YEASEN, 11201ES08). Each reaction was performed in technical triplicate. Primer sequences used in this study are provided in Supplementary Table 4.

### Immunofluorescence staining

Formalin-fixed, paraffin-embedded (FFPE) tissue sections were baked at 62 °C for 1 h, deparaffinized in xylene (3 × 20 min) (Sinopharm Chemical Reagent Co., Ltd, 10023418), and rehydrated through graded ethanol (Sinopharm Chemical Reagent Co., Ltd, 100092683) to water. Endogenous peroxidase activity was blocked with 3% H₂O₂ (Sinopharm Chemical Reagent Co., Ltd, 73113760) for 10 min, followed by PBS washes. Antigen retrieval was performed in 1 mmol/L Tris–EDTA buffer (pH 9.0) by boiling for 15 min and cooling to room temperature. Sections were blocked with 5% BSA for 20 min and incubated overnight at 4 °C with primary antibodies, including anti-NK1.1 antibody (1:50; Cell Signaling Technology, 39197) and anti-CD31 antibody (1:200; Cell Signaling Technology, 15585). After washing, the sections were incubated with HRP-conjugated goat anti-rabbit IgG secondary antibody (Abcam, ab6712) at 37 °C for 1 h. NK1.1 and CD31 were visualized using tyramide-conjugated fluorophores. For multiplex immunofluorescence staining, antigen retrieval was repeated between labeling cycles before incubation with the next primary antibody. Finally, slides were mounted with antifade medium containing DAPI (Cell Signaling Technology, 4083S).

### Single-cell RNA sequencing

Samples were minced with a sterile scalpel into ~1 mm³ fragments and suspended in 5 mL of digestion buffer consisting of 2 mg/mL collagenase type I (Sigma, C0130), 2 mg/mL collagenase type II (Sigma, C6885), and 200 U/mL DNase I (Worthington Biochemical Corporation, LS006344, Lakewood, USA) in RPMI medium (Corning, 10-040-CV). The suspension was incubated in a 37 °C water bath with shaking for 30 min, passed through a 100 µm cell strainer (Falcon, 352360, Corning Life Sciences, Tewksbury, MA, USA), and centrifuged at 400×*g* for 10 min at 4 °C. The cell pellet was resuspended in red blood cell lysis buffer (Solarbio, R1010, Beijing Solarbio Science & Technology Co., Ltd., Beijing, China), incubated for 2 min, passed through a 40 µm cell strainer (Falcon, 352340), and collected by centrifugation (400×*g*, 10 min, 4 °C). Non-NK immune cells were then depleted by negative magnetic selection using the EasySep™ Mouse NK Cell Isolation Kit (STEMCELL Technologies, 19855), followed by positive selection of CD45⁺ cells using the EasySep™ Mouse CD45 Positive Selection Kit (STEMCELL Technologies, 18945), according to the manufacturer’s instructions. After each centrifugation step (400×*g*, 10 min, 4°C), cells were manually counted using Trypan blue (Thermo, T10282) and AO-PI (LUNA, D23001, Logos Biosystems, Gunpo-si, Gyeonggi-do, Republic of Korea). The resulting single-cell suspensions were loaded onto a Chromium Controller (10x Genomics, Pleasanton, USA) and processed for single-cell 3′ gene expression profiling using the Chromium Next GEM Single Cell 3′ Kit v3.1 (10x Genomics, 1000268) and the Chromium Next GEM Chip G Single Cell Kit (10x Genomics, 1000120), following the manufacturer’s protocols. Briefly, single cells were encapsulated into gel beads-in-emulsion (GEMs), lysed, and the released RNA was barcoded by reverse transcription within individual GEMs. Cell-barcoded 3′ gene expression libraries were then generated and sequenced on an Illumina NovaSeq 6000 system (Illumina, San Diego, CA, USA) by Shanghai Biochip Co., Ltd. (Shanghai, China).

### ScRNA-seq data processing

Single-cell RNA-seq data were analyzed in R using Seurat (v5.4.0), DoubletFinder (v2.0.6), and Harmony (v1.2.4). Sequencing reads were aligned to the mouse reference genome mm39. Initial quality control was performed to remove low-quality or low-complexity cells. Specifically, cells with fewer than 200 detected genes (nFeature_RNA < 200), fewer than 500 UMIs (nCount_RNA < 500), more than 40,000 UMIs (nCount_RNA > 40,000), or more than 25% mitochondrial transcripts (percent.mt > 25) were excluded from downstream analysis.

To identify and remove potential doublets, each sample was first split according to orig.ident and processed independently. For each sample, data were normalized using NormalizeData, highly variable genes were identified using FindVariableFeatures with the “vst” method (nfeatures = 2000), and scaled using ScaleData. Principal component analysis was performed using RunPCA, followed by nearest-neighbor graph construction with FindNeighbors (dims = 1:30) and preliminary clustering with FindClusters (resolution = 0.1). UMAP dimensionality reduction was then performed using RunUMAP (dims = 1:30).

Potential doublets were identified using DoubletFinder. The optimal *pK* value was determined using paramSweep, summarizeSweep, and find.pK. The expected doublet rate was estimated based on cell loading density, and homotypic doublet proportions were modeled using modelHomotypic based on preliminary cluster assignments. Final doublet prediction was performed using doubletFinder with *pN* = 0.25, sample-specific *pK*, and the adjusted expected number of doublets. Only cells classified as singlets were retained for downstream analysis.

After doublet removal, all samples were merged for downstream analysis. Global clustering and cell type annotation were first performed based on canonical marker genes, after which NK cells were subsetted for focused re-analysis. For NK-cell subset analysis, data were normalized using log-normalization (normalization.method = “LogNormalize”, scale.factor = 10,000), and highly variable genes were identified using the “vst” method (nfeatures = 2000). Data were scaled using ScaleData with regression of orig.ident and percent.mt. Principal component analysis was performed, followed by batch-effect correction using Harmony with orig.ident as the grouping variable. Based on the Harmony reduction, nearest-neighbor graph construction was performed using FindNeighbors (dims = 1:20), followed by clustering with FindClusters (resolution = 0.3) and UMAP visualization using RunUMAP (dims = 1:20, reduction = “harmony”).

Cluster marker genes were identified using FindAllMarkers with only.pos = TRUE, min.pct = 0.25, and logfc.threshold = 0.5. Functional scores, including cytotoxicity and inflammatory signatures, were calculated using AddModuleScore based on predefined gene sets. Group comparisons were assessed using the Wilcoxon rank-sum test.

### Statistical analyses

All quantitative data are expressed as the mean ± standard deviation (SD), with individual measurements displayed. Statistical comparisons between two groups were performed using either an unpaired two-sided Student’s *t*-test or a Mann–Whitney *U* test, as appropriate based on data distribution and variance. For comparisons among multiple groups, one-way ANOVA followed by appropriate post hoc tests was used where applicable. Correlation analyses were carried out using Pearson’s correlation coefficient. Survival curves were generated using the Kaplan–Meier method, and differences were assessed by the log-rank test. Exact *P* values are reported in the figures, and results with *P* < 0.05 were considered statistically significant. Samples were excluded only in cases of technical failure, such as unsuccessful protein extraction. No samples were excluded based on the desired outcome. Investigators were not blinded to group allocation during outcome assessment.

## Results

### PCGF1 is upregulated in NSCLC and associated with poor prognosis

**Fig. 1:**
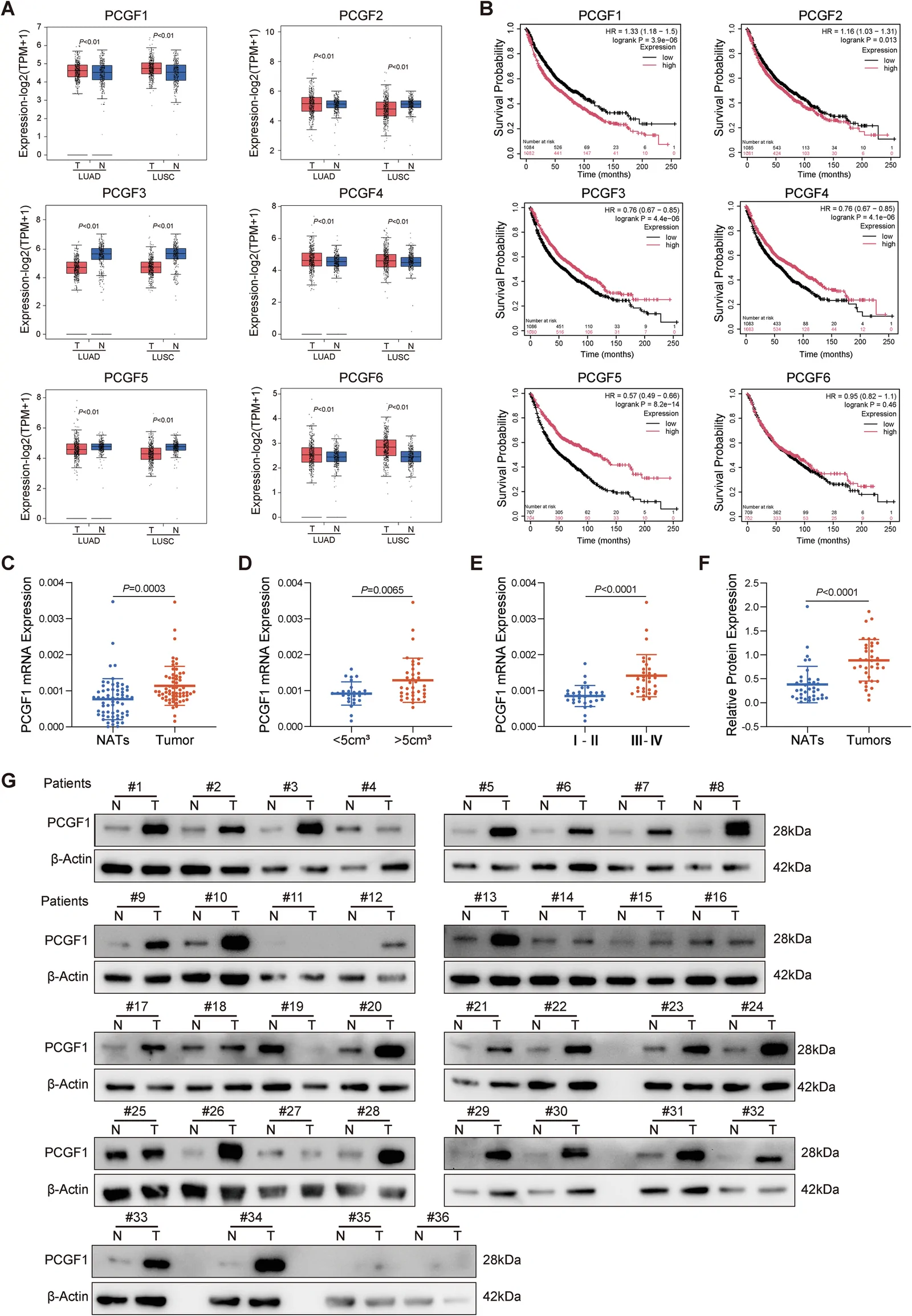
PCGF1 is upregulated in NSCLC and associated with poor prognosis. **A** Expression analysis of the PCGF gene family (PCGF1–6) in LUAD and LUSC based on the GEPIA platform. **B** Kaplan–Meier survival analysis of overall survival (OS) in patients with lung adenocarcinoma (LUAD) from the TCGA database. **C–E** Relative PCGF1 mRNA expression in clinical NSCLC samples from Huashan Hospital, Fudan University(*n* = 63). **F**, **G** Western blot analysis of 36 paired NSCLC and adjacent normal tissues(*n* = 36). Full and uncropped western blots corresponding to this figure are provided in Supplemental Material. Data are presented as mean ± SD with individual data points shown. Statistical significance was determined using the unpaired Student’s *t* test or the Mann–Whitney *U* test, depending on data distribution and variance. Exact *P* values are indicated in the figures.

The PCGF family constitutes an essential component of PRC1 and is indispensable for the deposition of H2AK119ub, thereby modulating bivalent promoters. To identify potential PCGF members involved in NSCLC, we first analyzed the expression profiles of PCGF1–6 in the TCGA cohort using the Gene Expression Profiling Interactive Analysis (GEPIA) platform. PCGF1, PCGF4, and PCGF6 were significantly upregulated in both lung adenocarcinoma (LUAD) and lung squamous cell carcinoma (LUSC) tissues compared with adjacent normal lung tissues (*P* < 0.05), whereas PCGF2, PCGF3 and PCGF5 showed no significant upregulation (Fig. 1A). Furthermore, Kaplan–Meier survival analysis revealed that high expression of PCGF1 and PCGF2 was significantly associated with shorter overall survival in NSCLC patients (Fig. 1B). Based on these findings, PCGF1 was selected as the primary focus for further investigation.

We next validated PCGF1 expression in clinical NSCLC specimens from Huashan Hospital, Fudan University, using RT-qPCR and Western blot. Consistent with the database analysis, PCGF1 expression was markedly elevated in tumor tissues compared with adjacent normal tissues (Fig. 1C, F, G). Higher PCGF1 levels were also detected in tumors with larger volume (>5 cm³), in advanced-stage (Ⅲ–Ⅳ) cases (Fig. 1D, E).

### PCGF1 suppresses NK cell-mediated immune responses in NSCLC

**Fig. 2:**
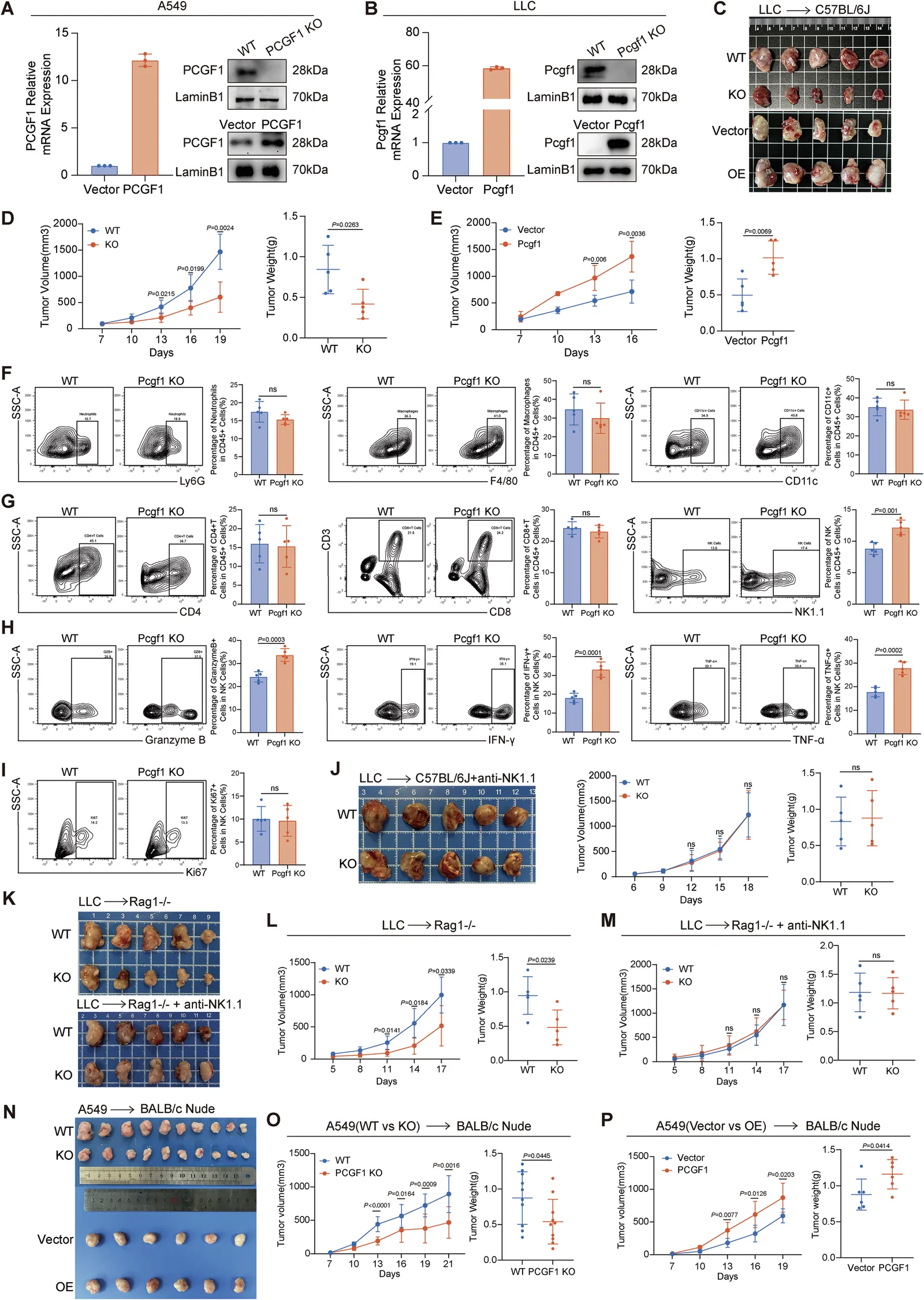
PCGF1 suppresses NK cell-mediated immune responses in NSCLC. **A** and **B** Verification of PCGF1 knock-out or overexpression efficiency in A549 or LLC cells. Relative PCGF1 mRNA and protein expression were analyzed by RT-qPCR (*n* = 3) and Western blot. LaminB1 was used as a loading control. Full and uncropped western blots corresponding to this figure are provided in Supplemental Material. **C** Tumor size in C57BL/6 J mice after WT, Pcgf1 KO, Vector and Pcgf1 OE LLC cell injection on the day of harvest. **D** and **E** Growth curves and tumor weights of subcutaneous LLC xenografts in C57BL/6J mice (*n* = 5, WT; *n* = 5, Pcgf1 KO; *n* = 5, Vector; *n* = 5, Pcgf1 OE). **F** Neutrophils, Macrophages, CD11c+ cells proportions in CD45+ cells (*n* = 5). **G** CD4⁺ T cells, CD8+ T cells, and NK cells proportions in CD45+ cells (n = 5). **H** Granzyme B+ cells, IFN-γ+ cells, TNF-α+ cells proportions in NK cells (*n* = 5). **I** Ki-67 + NK cells proportions in NK cells (*n* = 5). **J** Tumor size in C57BL/6J mice with anti-NK1.1 antibody-mediated NK cell depletion after WT, Pcgf1 KO LLC cell injection on the day of harvest. Tumor growth curves and tumor weights are shown (*n* = 5, WT; *n* = 5, Pcgf1 KO). **K–M** Tumor size in C57BL/6J Rag1^−/−^ mice with or without anti-NK1.1 antibody-mediated NK-cell depletion. Tumor growth curves and tumor weights are shown (without anti-NK1.1 antibody, *n* = 5 per group; with anti-NK1.1 antibody, *n* = 5 per group). **N** Tumor size in BALB/c nude mice after WT, PCGF1 KO, Vector, and PCGF1 OE A549 cell injection on the day of harvest. **O** and **P** Growth curves and tumor weights of subcutaneous A549 xenografts in BALB/c nude mice (*n* = 10, WT; *n* = 10, PCGF1 KO; *n* = 6, Vector; *n* = 6, PCGF1 OE). Data are presented as mean ± SD with individual data points shown. Statistical significance was determined using the unpaired Student’s *t* test or the Mann–Whitney *U* test, depending on data distribution and variance. Exact *P* values are indicated in the figures. ns, not significant (*P* ≥ 0.05).

To investigate the functional role of PCGF1 in NSCLC cells, we generated PCGF1-knockout (KO) A549 and LLC cells using the CRISPR-Cas9 system and established PCGF1-overexpressing (OE) stable lines via lentiviral transduction (Fig. 2A, B).

To evaluate the in vivo function of Pcgf1, we subcutaneously implanted Pcgf1 KO and Pcgf1 OE LLC cells into C57BL/6J mice and monitored tumor growth. Pcgf1 deletion markedly suppressed tumor growth, whereas Pcgf1 overexpression significantly promoted tumor progression, as reflected by both tumor volume and tumor weight (Fig. 2C–E).

Given that epigenetic therapies can enhance antitumor immunity by reprogramming tumor-intrinsic transcriptional states [6–9, 39], we next asked whether PCGF1 regulates immune-mediated cytotoxicity in the tumor microenvironment. Flow cytometric analysis of subcutaneous Pcgf1 KO LLC tumors showed no significant changes in the proportions or absolute numbers of neutrophils, macrophages, or CD11c+ cells between groups (Fig. 2F; Supplementary Fig. S1A, E–G). In contrast, both the proportion and absolute number of NK cells were increased in KO tumors, whereas CD4+ and CD8+ T cells remained unchanged (Fig. 2G; Supplementary Fig. S1B, H–J). Moreover, tumor-infiltrating NK cells in the KO group exhibited increased expression of IFN-γ, TNF-α, and Granzyme B, together with higher absolute numbers of these functional NK-cell subsets, indicating enhanced cytotoxic activity (Fig. 2H; Supplementary Fig. S1C, K–M). However, neither the proportion nor the absolute number of Ki-67+ NK cells was significantly altered (Fig. 2I; Supplementary Fig. S1D, N).

To determine whether the tumor-suppressive effect of Pcgf1 deficiency was causally dependent on NK cells, we performed additional in vivo validation using anti-NK1.1 antibody-mediated NK-cell depletion and Rag1^−/−^ mice, which lack T and B cells but retain NK cell function. The efficiency of NK cell depletion was confirmed by flow cytometry (Supplementary Fig. S1O, P). In wild-type C57BL/6J mice treated with anti-NK1.1 antibody, the growth difference between WT and Pcgf1 KO LLC tumors was largely abolished, with no significant difference in tumor volume or tumor weight (Fig. 2J). In contrast, in Rag1^−/−^ mice, Pcgf1 KO tumors still showed significantly reduced tumor growth and tumor weight compared with WT tumors, indicating that the antitumor effect of Pcgf1 deficiency was preserved in the absence of adaptive immune cells (Fig. 2K, L). Notably, further depletion of NK cells in Rag1^−/−^ mice abolished this growth-suppressive effect, and no significant difference in tumor volume or tumor weight was observed between WT and Pcgf1 KO tumors (Fig. 2K–M). Together, these results indicate that the tumor-suppressive effect of Pcgf1 deficiency is primarily dependent on NK cells, rather than on T/B cell-mediated adaptive immunity.

To further validate the pro-tumorigenic role of PCGF1 in human NSCLC cells, we subcutaneously implanted PCGF1 KO and PCGF1 OE A549 cells into BALB/c nude mice. Consistent with the results obtained in the LLC model, PCGF1 knockout suppressed tumor growth, whereas PCGF1 overexpression promoted tumor progression (Fig. 2N–P).

### PCGF1 deficiency shapes the transcriptional landscape and cytotoxic function of tumor-infiltrating NK cells

**Fig. 3:**
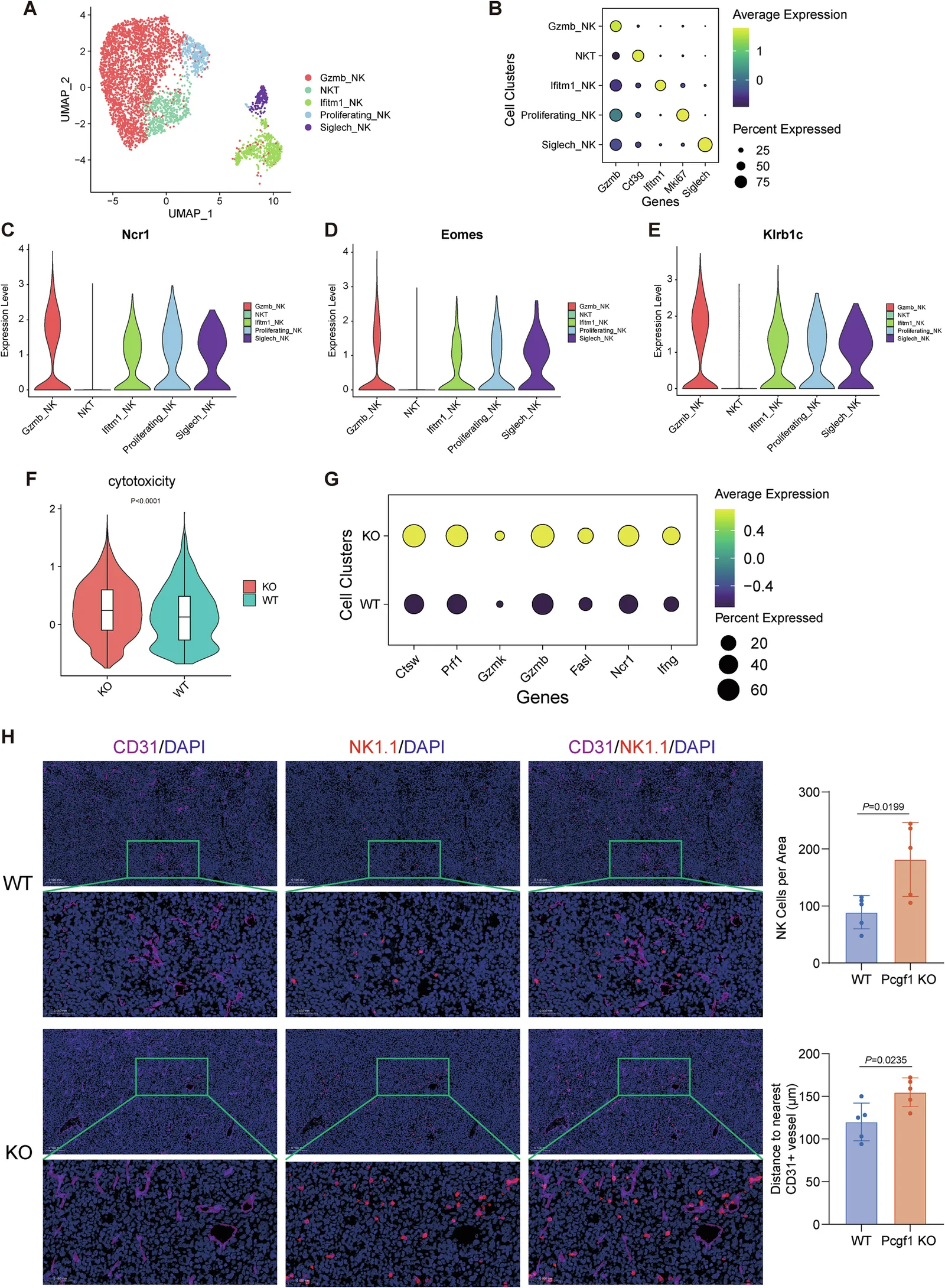
PCGF1 deficiency shapes the transcriptional landscape and cytotoxic function of tumor-infiltrating NK cells. **A** Uniform manifold approximation and projection (UMAP) of scRNA-seq of NK cells from tumors of WT and Pcgf1 KO LLC cells. Clusters are colored and annotated based on gene module score. **B** Dot plot showing the expression of representative marker genes (Gzmb, Cd3g, Ifitm1, Mki67, and Siglech) that define each cluster. **C–E** Violin plots of canonical NK-cell markers Ncr1, Eomes, and Klrb1c across clusters. **F** Comparison of NK-cell cytotoxicity scores between WT and Pcgf1 KO groups. **G** Dot plot illustrating the upregulation of NK effector genes in Pcgf1 KO relative to WT. **H** Representative images of immunofluorescence staining of CD31 (purple), NK1.1 (red), and DAPI (blue) in WT and Pcgf1 KO tumors. Main scale bars, 100 µm. Scale bars in insets, 50 µm. Quantification of average NK cell number and distance to the nearest CD31+ vessel per field of view, based on three independent images per tumor (*n* = 5 mice per group for WT and Pcgf1 KO). Data are presented as mean ± SD with individual data points shown. Statistical significance was determined using the unpaired Student’s *t* test or the Mann–Whitney *U* test, depending on data distribution and variance. Exact *P* values are indicated in the figures. ns, not significant (*P* ≥ 0.05).

To further investigate how PCGF1 shapes the transcriptional landscape of NK cells, we performed single-cell RNA sequencing on magnetically enriched tumor-infiltrating NK cells. Unsupervised clustering identified five major populations, designated Gzmb_NK, NKT, Ifitm1_NK, Proliferating_NK, and Siglech_NK (Fig. 3A), which were characterized by the marker genes Gzmb, Cd3g, Ifitm1, Mki67, and Siglech, respectively (Fig. 3B) [40, 41]. We next confirmed the robustness and purity of the NK-cell clusters by examining the canonical NK markers Ncr1, Eomes, and Klrb1c (Fig. 3C–E). Comparative analysis of NK cells between WT and Pcgf1 KO tumors revealed a significantly increased NK-cell cytotoxicity score in the Pcgf1 KO group (Fig. 3F), accompanied by a marked upregulation of NK effector genes (Fig. 3G), indicating that PCGF1 acts as a negative regulator of NK-cell effector function. Consistent with flow cytometry, immunofluorescence staining demonstrated a marked increase in NK cell infiltration in the Pcgf1 KO tumors (Fig. 3H).

### PCGF1 promotes evasion of NK cell-mediated cytotoxicity and inhibits NK cell migration

To investigate the interaction between NK cells and tumor cells, NK cells were isolated from the spleens of C57BL/6J mice, and >70% of the purified NK cells expressed Granzyme B, confirming high cytotoxic potential (Supplementary Fig. S2A, B).

**Fig. 4:**
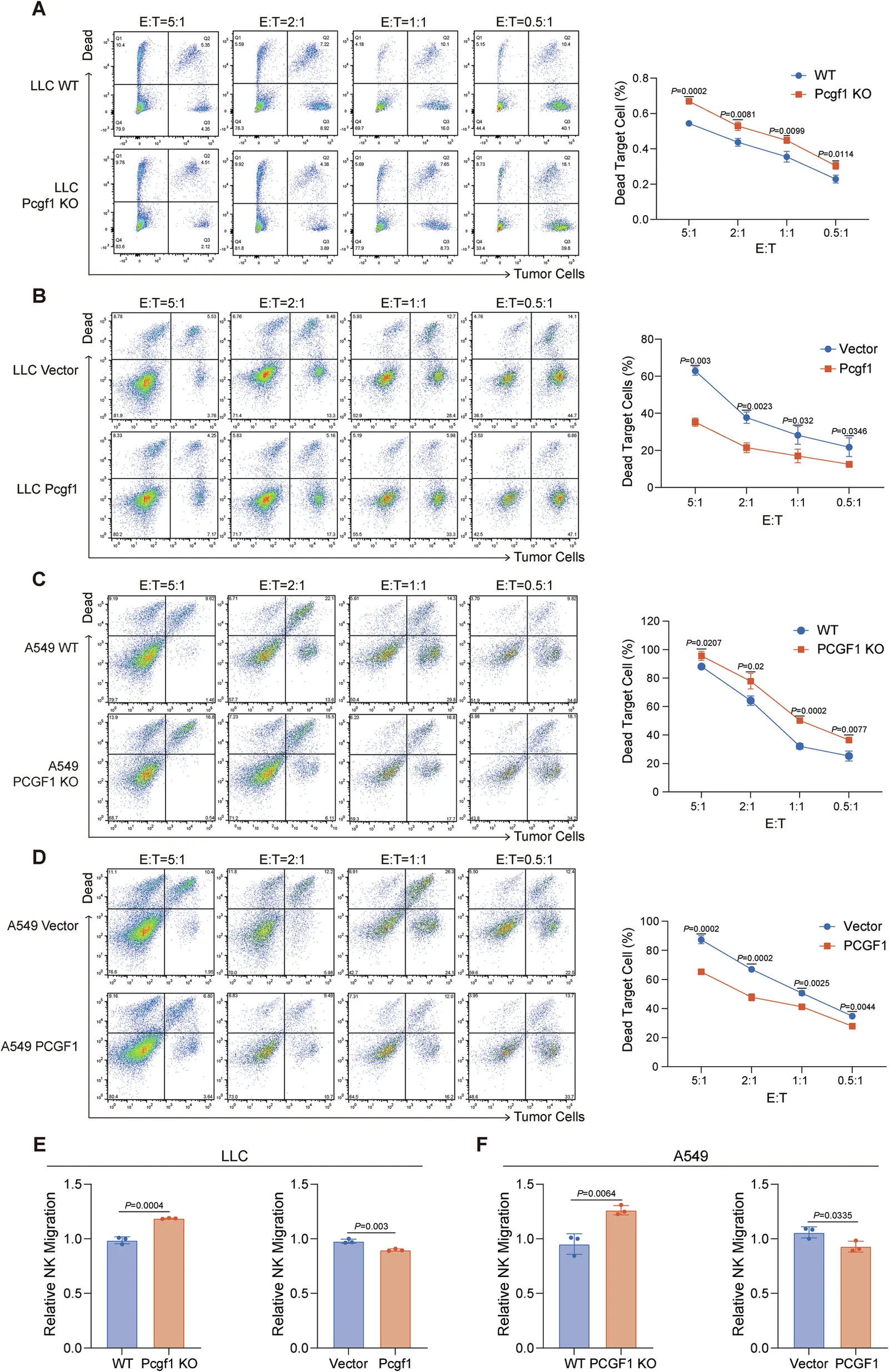
PCGF1 promotes evasion of NK cell-mediated cytotoxicity and inhibits NK cell migration. **A** and **B** Cytotoxicity assays in which Pcgf1 KO, Pcgf1 OE, and control LLC cells were co-cultured with NK cells at effector-to-target (E:T) ratios of 5:1, 2:1, 1:1, and 0.5:1 (*n* = 3). **C** and **D** Cytotoxicity assays in which PCGF1 KO, PCGF1 OE, and control A549 cells were co-cultured with NK-92MI cells at effector-to-target (E:T) ratios of 5:1, 2:1, 1:1, and 0.5:1 (*n* = 3). **E** and **F** Transwell migration assays showing the migratory response of NK cells (**E**) or NK-92MI cells (**F**) toward conditioned media derived from LLC (**E**) or A549 (**F**) cells with Pcgf1/PCGF1 knockout or overexpression (*n* = 3). Data are presented as mean ± SD with individual data points shown. Statistical significance was determined using the unpaired Student’s *t* test or the Mann–Whitney *U* test, depending on data distribution and variance. Exact *P* values are indicated in the figures. ns, not significant (*P* ≥ 0.05).

Pcgf1 KO and Pcgf1 OE LLC cells were co-cultured with NK cells at effector-to-target (E: T) ratios of 5:1, 2:1, 1:1, and 0.5:1. Under equivalent NK cell numbers and cytotoxic conditions, a significantly higher proportion of KO cells were killed compared with controls, whereas OE cells exhibited increased resistance to NK-mediated cytotoxicity (Fig. 4A, B). Similar results were obtained using A549 cells (Fig. 4C, D). In vitro migration assays showed that PCGF1 knockout promoted NK cell migration (Fig. 4E, F).

These findings indicate that high PCGF1 expression enables NSCLC cells to rapidly adapt to NK cell-induced immune pressure, thereby facilitating immune evasion, whereas loss of PCGF1 impairs this adaptive capacity and sensitizes tumor cells to NK cell-mediated killing.

### PCGF1 associates with chromatin bivalency in NSCLC cells

**Fig. 5:**
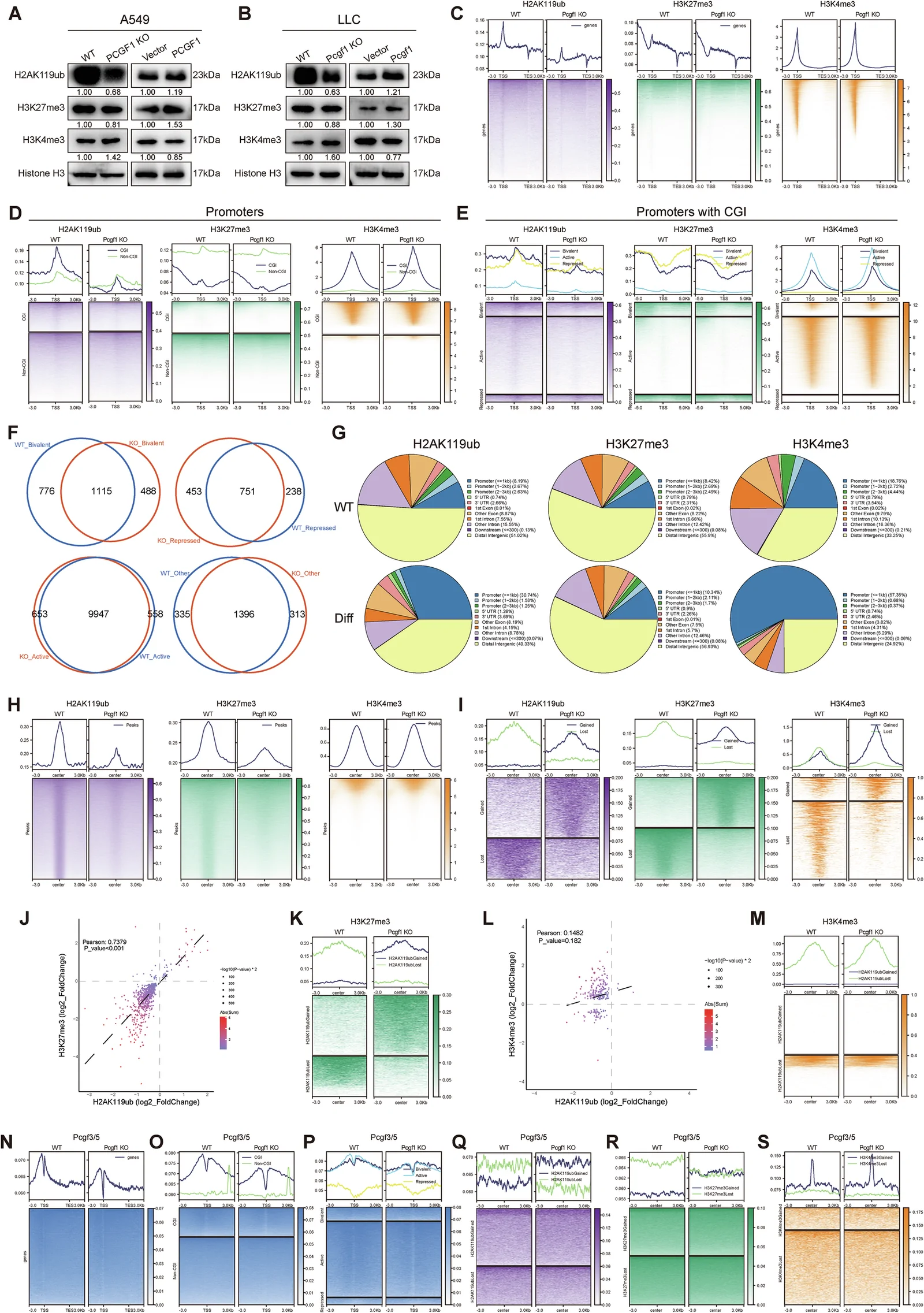
PCGF1 associates with chromatin bivalency in NSCLC cells. **A** and **B** Western blot analysis of H2AK119ub, H3K27me3, and H3K4me3 levels in A549 and LLC cells with PCGF1 KO or OE. Relative grayscale quantification is shown below the corresponding bands. Full and uncropped western blots corresponding to this figure are provided in Supplemental Material. **C** Heat maps of genome-wide enrichment profiles across gene bodies and ±3 kb around TSS and TES. **D** Enrichment of H2AK119ub, H3K27me3, and H3K4me3 at CGI and non-CGI promoters. **E** Average profiles of the three histone marks at bivalent, active, and repressed promoters. **F** Venn diagram comparing numbers of bivalent, active, repressed, and other promoters between WT and Pcgf1 KO cells. **G** Genomic annotation of H2AK119ub, H3K27me3, and H3K4me3 peaks. **H** Global CUT&Tag enrichment of H2AK119ub, H3K27me3, and H3K4me3 in Pcgf1 KO versus WT cells. **I** Differential peak analysis identifying significantly gained (log2FC > 1, padj < 0.05) and lost (log2FC < –1, padj < 0.05) regions for each histone mark, revealing locus-specific redistribution of chromatin modifications. **J** and **L** Correlation analyses between differential H2AK119ub and either H3K27me3 or H3K4me3 peaks at promoters (padj < 0.05). Abs = |H2AK119ub_log2FC | + |H3K27me3_log2FC | .Abs = |H2AK119ub_log2FC | +|H3K4me3_log2FC | . **K** H3K27me3 enrichment at H2AK119ub-gained regions and H2AK119ub-lost regions. **M** H3K4me3 enrichment at H2AK119ub-gained regions and H2AK119ub-lost regions. **N–P** Genome-wide Pcgf3/5 enrichment around TSS of gene bodies, CGIs, and different promoter classes in Pcgf1 KO and WT cells. **Q–S** Pcgf3/5 enrichment at regions with H2AK119ub-gained, H2AK119ub-lost, H3K27me3-gained, H3K27me3-lost, H3K4me3-gained and H3K4me3-lost regions. ns, not significant (*P* ≥ 0.05).

Described in 2006, bivalent domains silence developmental genes in ES cells while keeping them poised for activation [13]. We sought to investigate whether PCGF1, a key component of the PRC1.1, can influence bivalent promoters by regulating the deposition of H2AK119ub. We examined the global levels of H2AK119ub, H3K4me3, and H3K27me3 by Western blot in A549 cells with PCGF1 KO and OE. PCGF1 loss reduced H2AK119ub and H3K27me3, but increased H3K4me3, whereas PCGF1 overexpression had the opposite effect (Fig. 5A). The same pattern was observed in LLC cells (Fig. 5B).

To explore genome-wide changes, we performed Cleavage Under Targets and Tagmentation (CUT&Tag) for H2AK119ub, H3K27me3, and H3K4me3 in Pcgf1 KO and WT LLC cells. A heat map of genome-wide enrichment profiles across gene bodies and within ±3 kb of the transcriptional start sites (TSS) and transcriptional end sites (TES) showed decreased H2AK119ub and H3K27me3 enrichment at both the TSS and TES, whereas H3K4me3 enrichment increased around the TSS (Fig. 5C). CpG islands (CGIs) are generally associated with housekeeping genes [42–44]. CGIs are also associated with bivalent genes [45, 46]. Because H3K27me3 and H3K4me3 are predominantly enriched at gene promoters, especially CpG island (CGI) regions [13, 45–47], we investigated whether and how CGI-associated bivalent genes are regulated by sequence-specific recognition. In CGI, H2AK119ub and H3K27me3 enrichment around the TSS was decreased, whereas H3K4me3 enrichment was increased. In contrast, no significant differences were observed in non-CGI regions (Fig. 5D). We then classified CGI-associated promoters into bivalent, active, and repressed categories and analyzed the enrichment of the three histone marks across these promoter types [34]. H2AK119ub enrichment decreased in all three classes. H3K27me3 enrichment declined at bivalent and repressed promoters, while H3K4me3 increased at bivalent and active promoters (Fig. 5E). Next, we compared the numbers of bivalent, active, repressed, and other promoters between KO and WT cells. A Venn diagram illustrating the distribution of these promoter categories showed that all four promoter types were altered in KO cells relative to controls (Fig. 5F). We further annotated the genomic distributions of H2AK119ub, H3K27me3, and H3K4me3 peaks in WT cells, as well as the differential peaks between KO and WT cells for these histone modifications. In WT cells, 13.49% of H2AK119ub peaks were located at promoters, whereas 33.52% of the differential H2AK119ub peaks between KO and WT cells were promoter-associated. Similarly, 13.6% of H3K27me3 peaks in WT and 14.15% of the differential H3K27me3 peaks were located at promoters. In contrast, H3K4me3 showed a stronger promoter bias, with 25.92% of peaks in WT and 58.4% of the differential H3K4me3 peaks enriched at promoters (Fig. 5G).

The analysis revealed a global reduction of H2AK119ub and H3K27me3 enrichment accompanied by an increase in H3K4me3 relative to WT controls (Fig. 5H). Differential analysis identified significantly gained (log2FoldChange > 1, padj < 0.05) and lost (log2FoldChange < –1, padj < 0.05) peaks for each histone mark [48], revealing locus-specific rather than global redistribution (Fig. 5I). Since H2AK119ub levels changed at the TSS of bivalent, active, and repressed promoters (Fig. 5E), and because PCGF1-PRC1 mediated H2AK119ub is known to promote PRC2 recruitment and H3K27me3 stabilization [49], we investigated how differential H2AK119ub correlates with changes in H3K27me3 and H3K4me3. We correlated differential peaks at promoters (padj < 0.05) of H2AK119ub, H3K27me3, and H3K4me3. When H3K27me3 peaks were located within ±2 kb of differential H2AK119ub peaks, they were considered to affect the same loci. Plotting the log2FoldChange (log2FC) values revealed a strong positive correlation between H2AK119ub and H3K27me3 changes (Pearson’s *r* = 0.7379, *P* < 0.001) (Fig. 5J). Although no significant overall correlation was detected between H2AK119ub and H3K4me3, H3K4me3 alterations were mainly confined to genomic regions with decreased H2AK119ub deposition (Fig. 5L). Consistent with these results, heat maps showed that H3K27me3 was enriched at regions with H2AK119ub gained and depleted at H2AK119ub-loss sites (Fig. 5K), whereas H3K4me3 exhibited a mild increase at H2AK119ub-lost regions but was absent from H2AK119ub-gained loci (Fig. 5M).

While analyzing the relationship between differential H2AK119ub and H3K27me3 peaks, we noticed that a subset of these marks remained upregulated even after Pcgf1 KO (Fig. 5J). Previous studies in mouse embryonic stem cells (mESCs) have shown that PCGF3 and PCGF5 can recruit RING1B and EZH2 to promote the deposition of H2AK119ub and H3K27me3, and that PCGF3/5 colocalize with genomic regions enriched for H3K4me3 [16, 50]. To investigate this potential compensatory mechanism in NSCLC, we performed Chromatin immunoprecipitation sequencing (ChIP-seq) for Pcgf3/5 in the same cells. Heat map analysis revealed that Pcgf3/5 enrichment decreased around the TSS of gene bodies and within CGI regions (Fig. 5N, O). Moreover, Pcgf3/5 occupancy was reduced at bivalent and active promoters, but increased at repressed promoters (Fig. 5P). Peak analysis further showed that Pcgf3/5 enrichment increased at regions with H2AK119ub-gained and H3K27me3-gained peaks, but decreased at H2AK119ub-lost and H3K27me3-lost sites (Fig. 5Q, R). Similarly, Pcgf3/5 was elevated at H3K4me3-gained regions and reduced at H3K4me3-lost regions (Fig. 5S). These findings suggest that the unexpected increase in H2AK119ub and H3K27me3 after Pcgf1 loss may result from compensatory accumulation of Pcgf3/5 at these loci, potentially explaining the lack of correlation between H2AK119ub and H3K4me3.

It should be noted that, although our CUT&Tag and ChIP-seq analyses showed that genetic perturbation of PCGF1 was accompanied by significant changes in the global enrichment patterns of H2AK119ub, H3K27me3, and H3K4me3, the current evidence is still primarily based on omics-level correlations and is therefore insufficient to conclusively demonstrate a direct regulatory role of PCGF1 in chromatin bivalency. The present data accurately support the interpretation that PCGF1 is associated with chromatin bivalency modulation, or that PCGF1 knockout alters the landscape of bivalent histone marks in NSCLC cells. Further studies with stronger causal inference are required to validate the direct regulatory mechanism.

### PCGF1-dependent bivalent promoter remodeling reprograms tumor-intrinsic immune programs and NK cell responsiveness

**Fig. 6:**
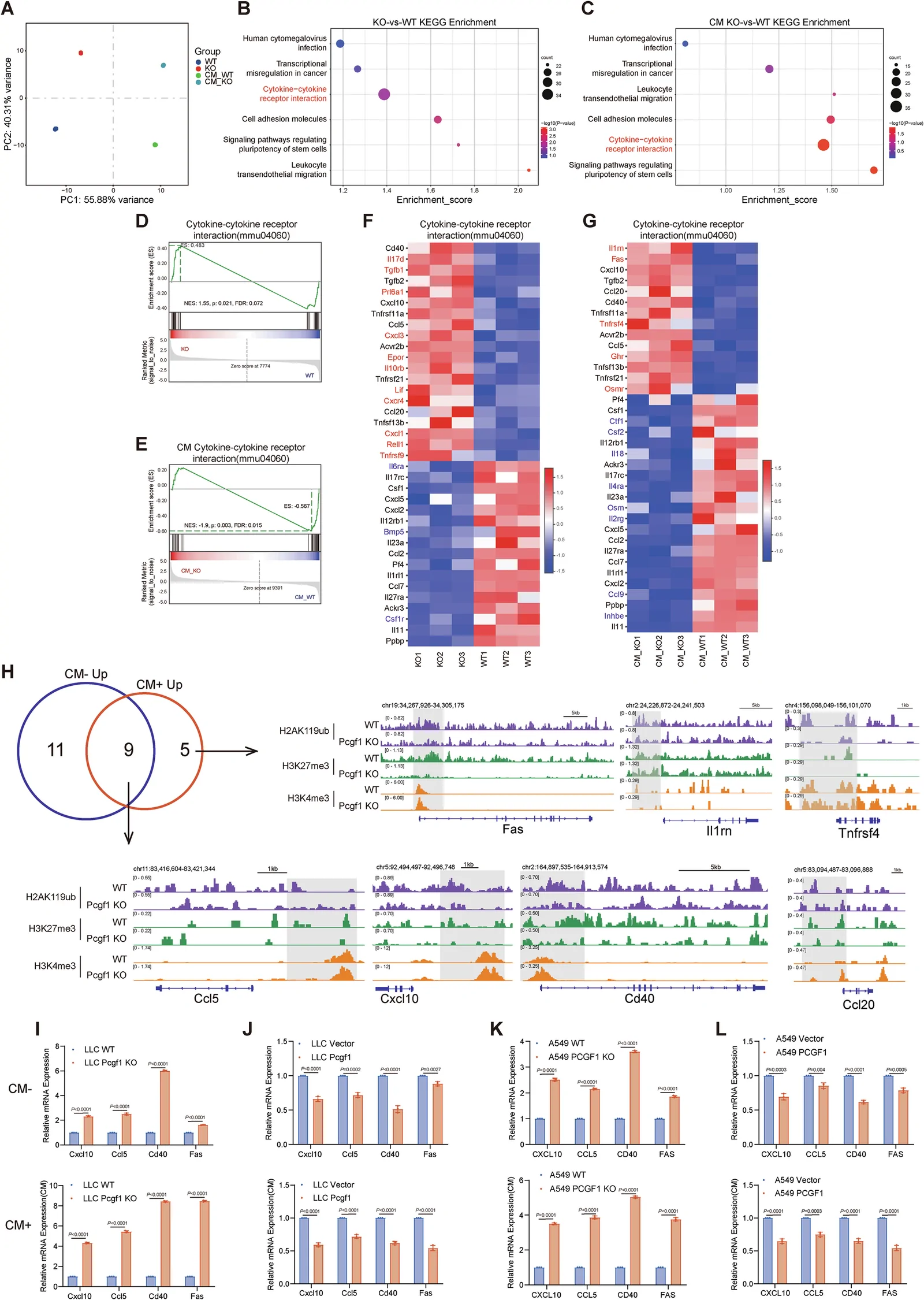
PCGF1-dependent bivalent promoter remodeling reprograms tumor-intrinsic immune programs and NK cell responsiveness. **A** PCA of RNA-seq profiles from WT and Pcgf1 KO LLC cells cultured in NK CM− or NK CM+. **B** and **C** KEGG pathway enrichment analysis of differentially expressed genes (DEGs, defined as genes with |log2FC | > 1 and adjusted *P* value < 0.05) in Pcgf1 KO versus WT cells under NK CM− (**B**) and NK CM+ (**C**). **D** and **E** GSEA of the cytokine-cytokine receptor interaction pathway in Pcgf1 KO versus WT cells under NK CM− (**D**) and NK CM+ (**E**). **F** and **G** Comparison of differentiation- and immune-related DEGs in Pcgf1 KO versus WT cells under NK CM− (**F**) and NK CM+ (**G**). **H** CUT&Tag profiles of H2AK119ub, H3K27me3, and H3K4me3 at the TSS of the DEGs. **I-L** RT-qPCR validation of Cd40, Cxcl10, Ccl5, and Fas in LLC and A549 cells cultured under two conditions (*n* = 3). Data are presented as mean ± SD with individual data points shown. Statistical significance was determined using the unpaired Student’s *t* test or the Mann–Whitney *U* test, depending on data distribution and variance. Exact *P* values are indicated in the figures. ns, not significant (*P* ≥ 0.05).

Described in 2006, bivalent domains silence genes while keeping them poised for activation [13]. To explore the transcriptional programs through which Pcgf1-dependent bivalent promoter remodeling influences NK cell responses, we cultured Pcgf1 KO and WT LLC cells under two conditions: standard medium (NK CM−) and NK cell-conditioned medium (NK CM+). Principal component analysis (PCA) showed clear transcriptomic separation among the four groups (Fig. 6A). To investigate the molecular changes induced by Pcgf1 loss, we performed KEGG pathway enrichment analysis on the differentially expressed genes. Genes involved in cytokine–cytokine receptor interaction, leukocyte migration, and transcriptional misregulation were significantly enriched (Fig. 6B, C). Consistently, gene set enrichment analysis (GSEA) showed a significant positive enrichment of the cytokine–cytokine receptor interaction pathway in Pcgf1 KO cells under standard medium (Fig. 6D), but a significant negative enrichment of the same pathway in Pcgf1 KO cells under NK CM+ (Fig. 6E), indicating a profound transcriptional rewiring between the two conditions.

We next compared the differentially expressed genes between the NK CM− and NK CM+ conditions (Fig. 6F, G). In these plots, genes uniquely upregulated in a given condition are shown in red, genes uniquely downregulated in blue, and genes altered in both conditions in black. This pattern indicates that Pcgf1-mediated epigenetic reprogramming not only reshapes the intrinsic transcriptional landscape of tumor cells but also reprograms their responsiveness to external NK-derived signals. In the NK CM- condition, 11 genes were uniquely upregulated, whereas 5 genes were uniquely upregulated under NK CM+. Nine genes were commonly upregulated in both conditions. At the transcription start sites (TSSs) of these differentially expressed genes, we observed reduced deposition of H2AK119ub and H3K27me3 together with altered H3K4me3 levels (either gain or loss), consistent with extensive remodeling of bivalent promoters (Fig. 6H) and tightly linking Pcgf1 loss-induced chromatin changes to the regulation of immune-related genes.

The genes commonly upregulated in Pcgf1 KO tumors under both conditions define a “core immune signature” and include Cd40, Cxcl10, and Ccl5. CD40 agonists (CD40a) activate macrophages, DCs, and NK cells and are critical for enhancing cross-presentation [51]. Immune cells can induce cell death in CD40-expressing cancer cells by triggering caspase-8 activation [52]. Tumor antigen-independent CD40 agonists, such as anti-CD40 antibodies, have been explored in therapeutic strategies to boost tumor immunity by, e.g., overcoming the tumor-induced immunosuppression [53–55]. Ectopic expression of CXCL10 in tumor cells promotes NK cell infiltration into tumors, which correlates with improved host survival [56]. CCL5 modulates the functional state and cytotoxic activity of local NK cells [57]. In contrast, genes commonly downregulated in both conditions included Csf1, Ccl2, Ccl7, Cxcl2, and Cxcl5, indicating that Pcgf1 KO tumors continuously suppress a CSF1/CCL2/7/CXCL2/5-centered myeloid/pro-tumor inflammatory axis regardless of NK CM exposure. Consistent with this, Csf1, Ccl2, and Cxcl5 have been associated with increased tumor growth and progression [58–60]. Genes specifically upregulated in Pcgf1 KO cells under NK CM+ included Fas, Tnfrsf4, and Il1rn. Fas is a canonical apoptosis-inducing death receptor of the TNF receptor (TNFR) superfamily [61, 62], suggesting that NK CM induces upregulation of Fas in Pcgf1 KO tumor cells and thereby renders them more susceptible to killing.

By contrast, genes uniquely upregulated in Pcgf1 KO cells under NK CM- included Il17d, Tgfb1, Prl6a1, Cxcl3, Cxcl1, Epor, Il10rb, Lif, Cxcr4, Rell1, and Tnfrsf9 (4-1BB), reflecting an intrinsic inflammatory and growth factor program in the absence of NK-derived cues. These differences were no longer significant upon exposure to NK CM, indicating that NK CM partially overrides this basal state. Taken together, Pcgf1 KO consistently upregulates key immune-recruiting factors such as Ccl5, Cxcl10, and Cd40, and, upon NK cell stimulation, further induces death receptors such as Fas, thereby increasing the susceptibility of KO tumor cells to NK cell-mediated cytotoxicity. Finally, RT-qPCR analysis confirmed the RNA-seq findings for representative genes (Fig. 6I–L).

### PCGF1 re-expression rescues epigenetic alterations and NK cell-related phenotypes in cells depleted of PCGF1

**Fig. 7:**
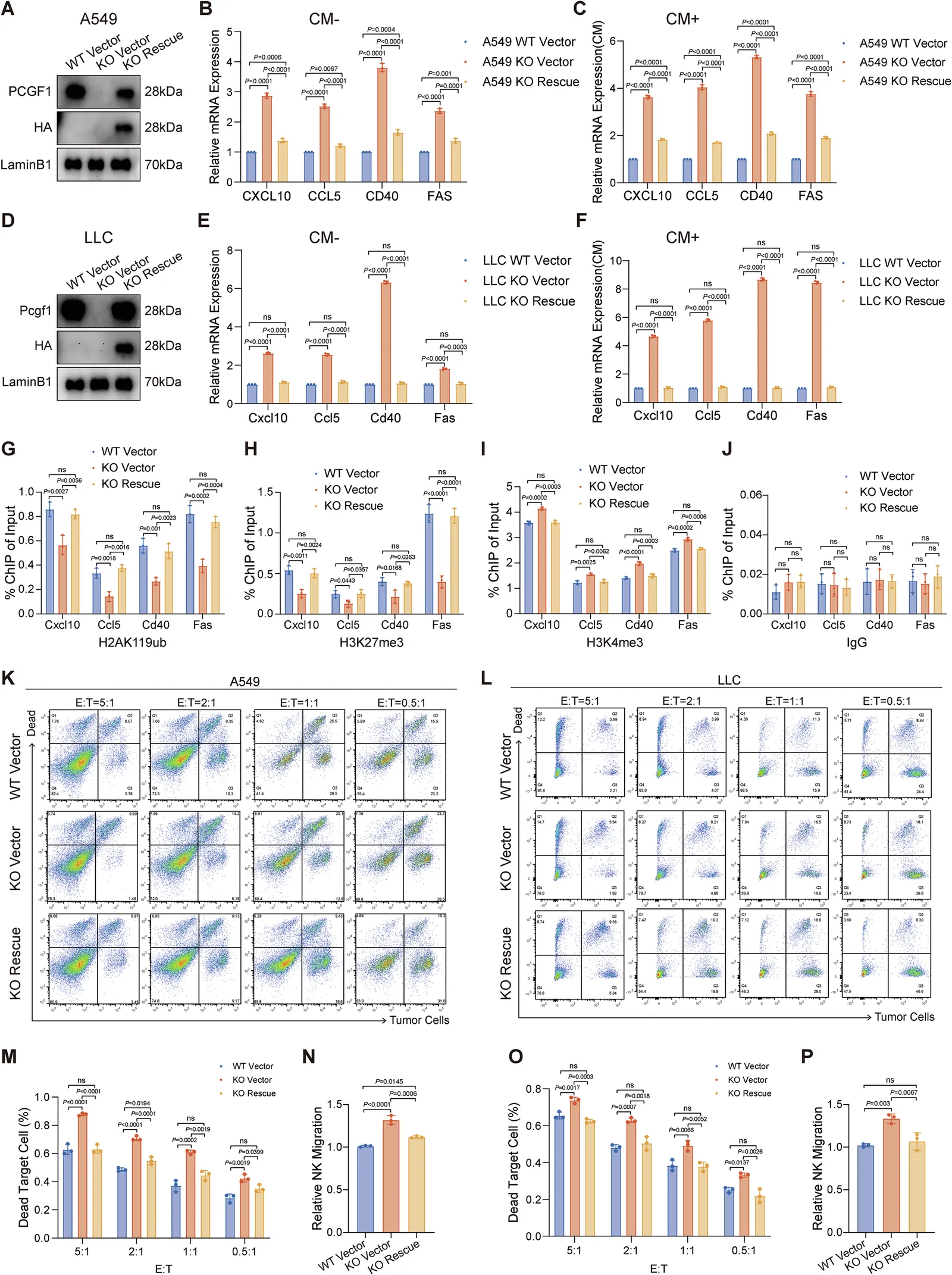
PCGF1 re-expression rescues epigenetic alterations and NK cell-related phenotypes in cells depleted of PCGF1. **A** and **D** Western blot analysis of PCGF1 re-expression in A549 and LLC Pcgf1-KO cells following lentiviral transduction. HA-tag indicates exogenous PCGF1 expression. Lamin B1 served as a loading control. Full and uncropped western blots corresponding to this figure are provided in Supplemental Material. **B** and **C** RT-qPCR analysis of CXCL10, CCL5, CD40, and FAS mRNA expression in A549 WT Vector, KO Vector, and KO Rescue cells under CM− and CM+ conditions (*n* = 3). **E** and **F** RT-qPCR analysis of Cxcl10, Ccl5, Cd40, and Fas mRNA expression in LLC WT Vector, KO Vector, and KO Rescue cells under CM− and CM+ conditions (*n* = 3). **G-J** ChIP-qPCR analysis of H2AK119ub (**G**), H3K27me3 (**H**), H3K4me3 (**I**), and IgG control (**J**) enrichment at the promoters of Cxcl10, Ccl5, Cd40, and Fas in LLC WT Vector, KO Vector, and KO Rescue cells (*n* = 3). **K** and **L** Representative flow cytometry plots showing NK cell-mediated cytotoxicity against A549 and LLC WT Vector, KO Vector, and KO Rescue cells at the indicated effector-to-target (E:T) ratios. **M** and **O** Quantification of dead target cells in A549 and LLC cytotoxicity assays shown in (**K**) and (**L**), respectively (*n* = 3). **N** and **P** Transwell migration assays showing NK cell recruitment toward A549 and LLC WT Vector, KO Vector, and KO Rescue cells (*n* = 3). Data are presented as mean ± SD with individual data points shown. Statistical significance was determined by one-way ANOVA followed by Tukey’s multiple comparisons test. Exact *P* values are indicated in the figures. ns, not significant (*P* ≥ 0.05).

To further assess whether the phenotypic and epigenetic alterations caused by PCGF1 loss could be reversed, we re-expressed PCGF1 in PCGF1-knockout A549 and LLC cells using a lentiviral system (Fig. 7A, D). Western blot showed that PCGF1 expression was partially restored in A549 KO cells, whereas its expression in LLC KO cells was restored to a level comparable to that in WT cells.

We next examined whether re-expression of PCGF1 could reverse the transcriptional changes induced by PCGF1 deficiency. RT-qPCR analysis showed that, both in the absence and presence of conditioned medium (CM), PCGF1 restoration in KO cells reversed the KO-induced transcriptional alterations of CXCL10, CCL5, CD40, and FAS in A549 cells (Fig. 7B, C). Similar results were observed in LLC cells, indicating that re-expression of Pcgf1 consistently counteracted the transcriptional effects caused by Pcgf1 loss (Fig. 7E, F).

To determine whether these transcriptional changes were accompanied by restoration of promoter-associated histone modifications, we performed ChIP-qPCR analysis in LLC WT Vector, KO Vector, and KO Rescue cells. At the promoters of four representative target genes (Cxcl10, Ccl5, Cd40, and Fas), Pcgf1 knockout altered the enrichment of H2AK119ub, H3K27me3, and H3K4me3, whereas re-expression of Pcgf1 largely reversed these changes, restoring the deposition pattern to a level close to that observed in WT Vector cells (Fig. 7G–J). These findings suggest that Pcgf1 influences H2AK119ub, H3K27me3, and H3K4me3 levels at the promoters of selected downstream target genes.

We further investigated whether restoration of PCGF1 could rescue NK cell-related phenotypes. In A549 cells, NK cell cytotoxicity assays showed that re-expression of PCGF1 partially restored the resistance of tumor cells to NK cell-mediated killing (Fig. 7K, M). Consistently, NK cell recruitment assays demonstrated that partial restoration of PCGF1 in A549 cells also partially reversed the enhanced NK cell recruitment caused by PCGF1 loss (Fig. 7N).

In LLC cells, restoration of Pcgf1 similarly rescued the functional consequences of Pcgf1 deficiency. Re-expression of Pcgf1 restored the ability of LLC cells to evade NK cell-mediated cytotoxicity and reduced NK cell recruitment compared with KO cells (Fig. 7L, O, and P). Together, these results indicate that re-expression of PCGF1 is sufficient to reverse, at least in part, the transcriptional, epigenetic, and NK cell-related phenotypes induced by PCGF1 loss in NSCLC cells.

## Discussion

Bivalent chromatin, marked by activating (H3K4me3) and repressive (H3K27me3) histone modifications, regulates key developmental genes and is frequently perturbed during tumorigenesis. Most work on bivalent promoters has focused on embryonic stem cells, where PRC1, PRC2, and Trithorax complexes coordinate lineage specification [49, 63]. How this circuitry is reconfigured in cancer to influence tumor-immune interactions has been less clear.

Here, we identify PCGF1, a core component of the PRC1.1 complex, as an epigenetic factor upregulated in NSCLC and associated with poor prognosis. CUT&Tag profiling demonstrates that PCGF1 controls a subset of bivalent promoters by modulating H2AK119ub deposition, which is tightly coupled to H3K27me3 enrichment at shared genomic loci. PCGF1 loss reduces both marks and coincides with context-dependent changes in H3K4me3, indicating that PCGF1 shapes the balance between activation and repression at dual-marked promoters. Genome-wide analyses further reveal that PCGF1 knockout redistributes bivalent, active, and repressive promoters and induces coordinated transcriptional changes in genes governing tumor progression and immune regulation.

Despite its positive role in H2AK119ub deposition, some loci exhibited increased H2AK119ub/H3K27me3 upon PCGF1 loss. ChIP-seq of PCGF3/5 suggests that PRC1.3/1.5 complexes compensate for PCGF1 deficiency at specific sites. Mechanistically, PCGF1 has been reported to be required for the implementation of H2AK119ub at its binding sites, whereas PCGF3/5 are required together for maintaining proper global H2AK119ub levels and X-chromosome inactivation in embryonic stem cells [16, 64, 65]. This distinction between locus-specific and global functions may help explain why the overall changes in H2AK119ub, and consequently H3K27me3, were relatively modest in our global analyses after PCGF1 knockout, despite clear promoter-associated redistribution revealed by chromatin profiling.

Interestingly, previous studies have also shown that PCGF3, PCGF5, and AUTS2 can activate transcription when forcibly recruited to chromatin in mESCs and HEK293T cells [16, 66]. Consistent with this, ChIP-seq data revealed that PCGF3 and PCGF5 co-localize with genomic regions enriched for H3K4me3, a histone mark associated with actively transcribed promoters, while being depleted from regions enriched for H3K27me3 and H2AK119ub [16, 67]. This is in line with our observation of compensatory PCGF3/5 occupancy in ChIP-seq analyses after PCGF1 loss, and may partly explain why the global changes in H2AK119ub, H3K27me3, and H3K4me3 were not highly pronounced. Together, these findings suggest that different PRC1 subcomplexes may exert distinct and even partially opposing functions in tumor cells, with PCGF1 being more closely linked to repression at selected promoters, whereas PCGF3/5 may contribute both to global chromatin regulation and, in certain contexts, transcriptional activation.

This possibility is also intriguing in light of our clinical observations. In NSCLC, PCGF1 is upregulated in tumor tissues and its high expression is associated with poor prognosis, whereas PCGF5 is downregulated in tumors relative to adjacent normal tissues, and higher PCGF5 expression is significantly associated with better patient outcomes. Based on the known functions of these PRC1 family members, we speculate that this contrast may reflect their distinct biological roles in NSCLC. Higher PCGF5 expression, given its reported association with transcriptional activation and active chromatin, may be linked to a less aggressive tumor state or to activation of gene programs that restrain tumor progression. However, the precise transcriptional targets and pathways regulated by PCGF5 in NSCLC remain unclear. It will therefore be important in future studies to determine which genes and signaling pathways are activated by PCGF5, how PCGF5 contributes to global H2AK119ub regulation in tumor cells, and whether its downstream effects are related to tumor immune regulation or to other malignant phenotypes such as proliferation, survival, or metastasis. Since the present study mainly focused on the promoter-associated epigenetic functions of PCGF1, these interesting aspects of PCGF5 warrant further investigation.

Immunotherapy has transformed cancer treatment, but is limited by resistance and suboptimal responses. Epigenetic modifiers are emerging as attractive combinatorial targets, and PRC2-mediated silencing of the MHC-I pathway is known to promote immune escape from cytotoxic T cells [68, 69]. Our data extend this paradigm to PRC1.1: integrating single-cell RNA-seq, flow cytometry, and immunofluorescence, we show that PCGF1 loss enhances NK cell infiltration and cytotoxicity in NSCLC. Mechanistically, PCGF1-dependent H2AK119ub/H3K27me3 at bivalent promoters represses CCL5, CXCL10, CD40, FAS, and other immune genes, dampening cytokine-receptor signaling in tumor cells, impairing NK cell recruitment and effector function, and reducing Fas-mediated apoptosis. PCGF1 knockout relieves this repression, restores chemokine and death receptor expression, and reinforces NK cell-mediated anti-tumor immunity.

These findings suggest that PCGF1 may represent a promising epigenetic target in NSCLC. Since PCGF1 contributes to NK cell immune evasion by suppressing the expression of key immune factors in tumor cells, it would be of considerable interest to test whether PCGF1 inhibition can be combined with NK cell-activating therapies to achieve enhanced antitumor effects in NSCLC models. In addition, given that PCGF1 is highly expressed in NSCLC and associated with poor prognosis, its expression level may also have potential value as a predictive biomarker for response to NK cell-oriented immunotherapy.

## Supplementary information

Supplementary Figures

Original images of western blot

Supplementary File

Supplementary Table 1

Supplementary Table 2

Supplementary Table 3

Supplementary Table 4

## Data Availability

The raw bulk RNA-seq, CUT&Tag, and ChIP-seq sequencing data generated in this study have been deposited in the Genome Sequence Archive (GSA) under BioProject accession number PRJCA062005. The bulk RNA-seq data are publicly accessible under accession number CRA041292, the CUT&Tag data under CRA041279, and the ChIP-seq data under CRA041297. Processed single-cell RNA-seq data have been deposited in OMIX under accession number OMIX016194. All other datasets generated or analyzed during this study can be obtained from the corresponding authors upon reasonable request.

## Code availability

This study did not generate original codes. All software and algorithms used in this study are publicly available and listed in the corresponding “Methods” section. Any additional information required to reanalyze the data reported in this paper is available from the corresponding author upon request.
