# Supplementary material for: PCGF1-mediated bivalent promoter remodeling enables NK cell immune evasion in non-small cell lung cancer
Source: Cell Death Dis. 2026 Jun 30;17(1):811. doi: 10.1038/s41419-026-09003-6 (PMC13586340; doi:10.1038/s41419-026-09003-6)
Supplement: Supplementary file 1 [file 41419_2026_9003_MOESM1_ESM.docx]

Supplementary Figures

PCGF1-Mediated Bivalent Promoter Remodeling Enables NK Cell Immune Evasion in Non-Small Cell Lung Cancer

Hui Zhou ^a, b,1^, Hechun Lin ^c,1^, Yiru Kong ^a, 1^, Hongyu Pan ^d^, Wenjun Chai ^d^, Lei Sun ^d^, Yu Wang ^a^, Mengxi Ge ^a^, Xiaoyu Ji ^a^, Rongrong Yao ^a^, Qing Wang ^a^, Tao Liu ^a^, Mingxia Yan ^d^, Xiaohua Liang ^a^, Hong Li ^c, *^, Jing Li ^a, *^, Xinli Zhou ^a, *, 2^

**Supplementary Fig. S1. PCGF1 suppresses NK cell–mediated immune responses in NSCLC**

**A-D** Gating strategies used to identify myeloid and lymphoid cell subsets, cytokine-producing NK cells and Ki-67+ NK cells. **E-G** Absolute numbers of neutrophils, macrophages and CD11c+ cells, calculated from a total acquisition count of 3 × 10^5^ events. **H-J** Absolute numbers of CD4+ T cells, CD8+ T cells and NK cells, calculated from a total acquisition count of 1.1 × 10^5^ events. **K-M** Absolute numbers of Granzyme B+ NK cells, IFN-γ+ NK cells and TNF-α+ NK cells, calculated from a total acquisition count of 8.3 × 10^4^ events. **N** Absolute number of Ki-67+ NK cells, calculated from a total acquisition count of 4 × 10^5^ events. **O, P** The efficiency of NK-cell depletion of C57BL/6J and Rag1^-/-^ in spleens.

**Supplementary Fig. S2. PCGF1 promotes evasion of NK cell–mediated cytotoxicity and inhibits NK cell migration**

**A** Flow cytometric analysis of NK cells isolated from the spleens of C57BL/6J mice showing that more than 70% of the purified NK cells expressed Granzyme B, confirming high cytotoxic activity. **B** Diagram illustrating the gating strategy used for flow cytometry analysis.
