# Supplementary material for: PCGF1-mediated bivalent promoter remodeling enables NK cell immune evasion in non-small cell lung cancer
Source: Cell Death Dis. 2026 Jun 30;17(1):811. doi: 10.1038/s41419-026-09003-6 (PMC13586340; doi:10.1038/s41419-026-09003-6)
Supplement: Supplementary file 3 [file 41419_2026_9003_MOESM3_ESM.docx]

**Supplementary File. Codon-optimized coding sequences used for rescue constructs**

**PCGF1-Mediated Bivalent Promoter Remodeling Enables NK Cell Immune Evasion in Non-Small Cell Lung Cancer**

Mouse Pcgf1-HA codon-optimized CDS (5′–3′)

ATGAGACTGAGAAATCAGCTGCAGAGCGTGTACAAGATGGACCCCCTGAGAAACGAGGAAGAGGTGAGAGTGAAGATCAAGGACCTGAACGAGCACATCGTGTGCTGCCTGTGCGCCGGCTACTTCGTGGACGCCACCACCATCACCGAGTGCCTGCACACCTTCTGCAAGAGCTGCATCGTGAAGTACCTGCAGACAAGCAAGTACTGCCCCATGTGCAACATCAAGATCCACGAGACACAGCCCCTGCTGAACCTGAAGCTGGACAGAGTCATGCAAGACATCGTGTACAAGCTGGTGCCCGGCCTGCAAGACAGCGAGGAGAAGAGGATTAGAGAGTTCTATCAGAGCAGAGGCCTGGACAGAGTGAGCCAACCCTCCGGCGAAGAACCTGCCCTGAGCAACCTGGGCCTGCCCTTCAGCAGCTTCGACCACAGCAAGGCCCACTACTACAGATACGACGAGCAGCTGAGCCTGTGCCTGGAGAGACTGAGCAGCGGCAAGGACAAGAACAAGAACGTGCTGCAGAACAAGTACGTGAGATGCAGCGTGAGAGCCGAGGTGAGACACCTGAGAAGAGTGCTGTGCCACAGACTGATGCTGAACCCTCAGCACGTGCAGCTGCTGTTCGACAACGAGGTGCTGCCCGACCACATGACCATGAAGCAGCTGTGGCTGAGCAGATGGTTCGGCAAGCCTAGCCCCCTGCTCCTGCAGTACAGCGTGAAGGAGAAGAGAAGAGGCAGCGGCTACCCCTACGACGTGCCCGACTACGCCTAA

Human PCGF1-HA codon-optimized CDS (5′–3′)

ATGGCTAGCCCCCAAGGCGGGCAGATCGCCATCGCCATGAGACTGAGAAATCAGCTGCAGAGCGTGTACAAGATGGACCCCCTGAGAAACGAGGAAGAGGTGAGAGTGAAGATCAAGGACCTGAACGAGCACATCGTGTGCTGCCTGTGCGCCGGCTACTTCGTGGACGCCACCACCATCACCGAGTGCCTGCACACCTTCTGCAAGAGCTGCATCGTGAAGTACCTGCAGACAAGCAAGTACTGCCCCATGTGCAACATCAAGATCCACGAGACACAGCCCCTGCTGAACCTGAAGCTCGATAGGGTGATGCAAGACATCGTGTACAAGCTAGTGCCCGGCCTGCAAGACAGCGAGGAGAAGAGGATCAGAGAGTTCTATCAGAGCAGAGGCCTGGACAGAGTGACACAGCCCACCGGCGAAGAGCCTGCCCTGAGCAACCTGGGCCTGCCCTTCAGCAGCTTCGACCACAGCAAGGCCCACTACTACAGATACGACGAGCAGCTGAACCTGTGCCTGGAGAGACTGAGCAGCGGCAAGGACAAGAACAAGAGCGTGCTGCAGAACAAGTACGTGAGATGCAGCGTGAGAGCCGAGGTGAGACACCTGAGAAGAGTGCTGTGCCACAGACTGATGCTGAACCCTCAGCACGTGCAGCTGCTGTTCGACAACGAGGTGCTGCCCGACCACATGACCATGAAGCAGATCTGGCTGAGCAGATGGTTCGGCAAGCCTAGCCCCCTGCTCCTGCAGTACAGCGTGAAGGAGAAGAGAAGAGGCAGCGGCTACCCCTACGACGTGCCCGACTACGCCTGA
