# Supplementary material for: PCGF1-mediated bivalent promoter remodeling enables NK cell immune evasion in non-small cell lung cancer
Source: Cell Death Dis. 2026 Jun 30;17(1):811. doi: 10.1038/s41419-026-09003-6 (PMC13586340; doi:10.1038/s41419-026-09003-6)
Supplement: Supplementary file 4 [file 41419_2026_9003_MOESM2_ESM.docx]

**Original images of western blot**
